# Building de novo cryo-electron microscopy structures collaboratively with citizen scientists

**DOI:** 10.1371/journal.pbio.3000472

**Published:** 2019-11-12

**Authors:** Firas Khatib, Ambroise Desfosses, Foldit Players, Brian Koepnick, Jeff Flatten, Zoran Popović, David Baker, Seth Cooper, Irina Gutsche, Scott Horowitz

**Affiliations:** 1 Department of Computer and Information Science, University of Massachusetts Dartmouth, Dartmouth, Massachusetts, United States of America; 2 Institut de Biologie Structurale, University Grenoble Alpes, CEA, CNRS, Grenoble, France; 3 Department of Biochemistry, University of Washington, Seattle, Washington, United States of America; 4 Center for Game Science, Paul G. Allen School of Computer Science and Engineering, University of Washington, Seattle, Washington, United States of America; 5 Khoury College of Computer Sciences, Northeastern University, Boston, Massachusetts, United States of America; 6 Department of Chemistry and Biochemistry and the Knoebel Institute for Healthy Aging, University of Denver, Denver, Colorado, United States of America

## Abstract

With the rapid improvement of cryo-electron microscopy (cryo-EM) resolution, new computational tools are needed to assist and improve upon atomic model building and refinement options. This communication demonstrates that microscopists can now collaborate with the players of the computer game Foldit to generate high-quality de novo structural models. This development could greatly speed the generation of excellent cryo-EM structures when used in addition to current methods.

## Abstract 2

This Community Page article demonstrates that microscopists can now collaborate with the players of the computer game Foldit to generate thorough high-quality de novo structural models; this development could greatly speed the generation of excellent Cryo-EM structures when used as a complement to current methods.

## Main text

Less than a decade ago, before the “resolution revolution,” cryo-electron microscopy (cryo-EM) was indulgently called “blobology” [1–4]. Whereas seminal work of cryo-EM experts resulted in high-resolution 3D maps and atomic models of ordered assemblies such as 2D crystals, helical arrays, and icosahedral viruses [5–11], commonly obtained 3D maps of less regular or asymmetric objects could be interpreted only in terms of global 3D architecture, domain organization, and—at most—secondary structure elements. Atomic model building was the privilege and expertise of crystallographers, requiring careful consideration of structural details such as bond geometry, steric clashes, and hydrogen bonds. Now, however, thanks to spectacular progress in both hardware and software, cryo-EM scientists suddenly face the necessity of building atomic models into near-atomic resolution maps. This unanticipated promotion from “blobologists” to “structure solvers” [12] is not as straightforward as it may seem, because model building and refinement are labor-intensive and require expertise in macromolecular structure. Spurred on by the improved resolution of newly obtained maps, the growing cryo-EM community has generated hundreds of excellent—but also some error-containing and energetically unfavorable—atomic models [1,13,14]. Such errors not only jeopardize the cryo-EM field itself but also misguide downstream research that relies on accurate molecular models, such as mutational analysis and structure-based drug design.

Although rigorous structure and model validation tools tailored for cryo-EM are currently under intense development [14,15], improving the quality of cryo-EM model building remains an important area of research. The recent introduction of computational model-building tools geared toward cryo-EM offer the possibility of automated model building [16–19]. However, building accurate models into near-atomic resolution cryo-EM maps remains a substantial challenge, because atom positions at this resolution are not unambiguous and must be inferred with aid from molecular mechanics models.

Citizen scientists have been able to contribute to challenging problems in fields such as RNA design [20], neuroscience [21], sequence alignment [22], and quantum physics [23]. Thus, one possible model-building option is Foldit (https://fold.it), a citizen science computer game that challenges players to solve complex biochemistry puzzles [24]. Recent improvements to Foldit enable players to build protein structures into crystallographic, high-resolution maps more accurately than expert crystallographers or automated model-building algorithms [25]. Unlike crystallographic maps, which often rely on phase data inferred from model coordinates, cryo-EM maps are more suitable targets for Foldit because averaged EM data are directly interpretable and are independent of the model. Here, we show that crowd-powered model building by Foldit players can indeed substantially help cryo-EM scientists.

To assess the usefulness of Foldit for cryo-EM, the players were provided with cryo-EM densities corresponding to 4 segmented subunits of the antefeeding prophage (AFP, from a soil bacterium *Serratia entomophila*)—Afp1, Afp5, Afp7, and Afp9 [34]. For ease of comparison, the maps were filtered to 3.2-Å resolution to avoid local quality variation and contain information up to the same resolution of 3.2 Å, which is currently considered fairly high by the cryo-EM community but still arduously low for fully automated model-building algorithms. The players tried to achieve the highest possible Foldit score, which combines the Rosetta force field with map fitting [26,27]. The structures generated by players were compared with those produced by a cryo-EM expert who created models using the manual model-building and real-space refinement software Coot [28], followed by additional real-space refinement in Phenix [29]. Structures generated by the state-of-the-art automated model-building algorithms Rosetta “denovo_density,” Phenix Map-to-Model, ARP/wARP, and Buccaneer [16–19] were included in the comparison. Standard EM validation tools and crystallographic statistics were used to evaluate the 4 approaches.

**Fig 1:**
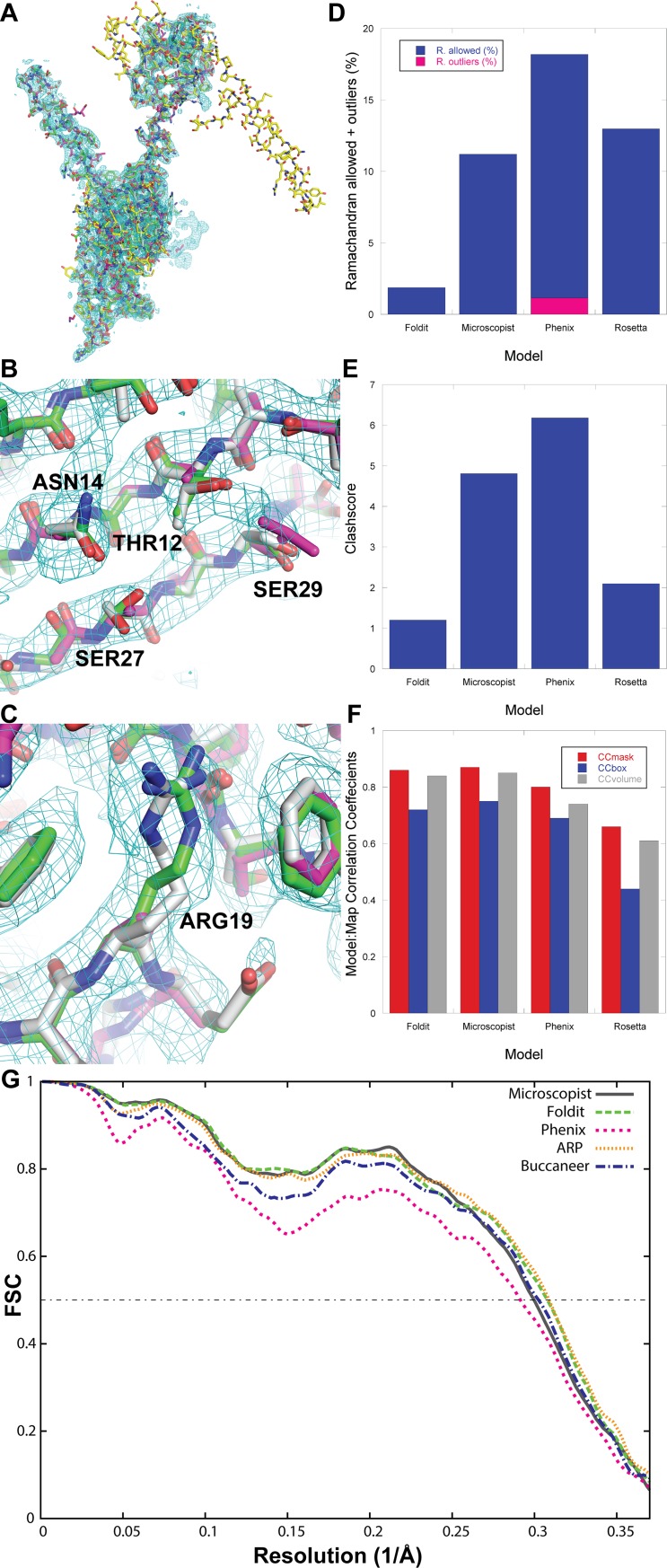
Comparison of model building for Afp7 in (A) an overall view and (B and C) views to compare side-chain fitting. The Foldit structure is rendered in green, the microscopist structure in gray, the Phenix model in magenta, and Rosetta model in yellow. Because of the large deviations from the other structures, the Rosetta model is omitted in the zoomed-in views in parts B and C. The electron potential map is contoured at 2 *σ*. (D, E, and F) Comparison of key geometric and map fit parameters for each of the cases displayed here. (D) Comparison of Ramachandran outlier and allowed backbone conformations. (E) Comparison of Molprobity Clashscore—in both cases, lower is better. (F) Comparison of 3 different map-to-model correlation coefficients, in which higher values are better. More complete statistical analysis can be found in Table 1 and S8 Fig, with the underlying data provided in S1 Data. (G) Map-to-model FSC curves for Microscopist (gray), Foldit (green), Phenix (pink), ARP w/ARP (orange), and Buccaneer (blue) models. CC_mask_, CC_box_, CC_peaks_, and CC_volume_ are correlation coefficients calculated between the model and the map. The differences between these correlation coefficients arise from whether the entire map is used (CC_box_), only the map around the atomic centers (CC_mask_), the molecular envelope defined by the model (CC_volume_), or the strongest peaks in the model and map (CC_peaks_) [14]. Afp7, antefeeding prophage 7; FSC, Fourier shell correlation; RMSD, root mean square deviation.

Table 1 compares the results of the various methods, using multiple criteria to evaluate both the model fit to the map and physical plausibility. Rosetta, Phenix, and Buccaneer struggled to correctly place certain chains in the appropriate density (Fig 1, S1–S11 Figs). This difficulty likely stems from errors in side-chain assignment, because the map resolution is often too poor for unambiguous side-chain identification, and these approaches fit regions of the map with incorrect sequences (Fig 1). All 4 automated methods had difficulty generating plausible geometry (Table 1, S1 Text). The Foldit structures and those generated by the microscopist produced accurate structures that were geometrically plausible and fit the maps well. Examining the models more closely shows that in most cases, the Foldit players placed slightly greater importance on bond geometry and steric clashes than the microscopist, who sacrificed these aspects for better fitting to the map (Table 1, Fig 1, S1 Text, S1 Table, and S1–S3 Figs). Although close in quality, at this resolution, we suggest it is appropriate to prioritize model geometry over map fit (Fig 1D–1G). Indeed, above 3-Å resolution, outliers are unlikely to be sufficiently supported by experimental data [14].

**Table 1:** Validation scores for all models.

Protein	Model	CC_mask_	CC_box_	CC_peaks_	CC_volume_	FSC Average	Molprobity Score	Clash Score	Rama. Favored (%)	Rama. Allowed (%)	Rama. Outliers (%)	CaBLAM Outliers	RMSD B Length (Å)	RSMD Angles (°)
Afp1	Foldit	0.9	0.64	0.67	0.89	0.51	0.5	0	98.64	1.36	0	0.69	0.008	0.684
	Microscopist	0.93	0.68	0.72	0.91	0.53	1.52	3.52	94.52	4.11	1.37	2.78	0.008	0.872
	ARP/wARP	0.92	0.68	0.72	0.90	0.53	1.80	7.66	94.37	4.93	0.70	2.14	0.007	0.955
	Phenix	0.85	0.63	0.67	0.81	0.44	1.83	6.17	91.75	8.25	0	0	0.006	0.754
	Buccaneer	0.90	0.61	0.66	0.87	0.51	1.60	2.73	90.51	9.49	0	5.88	0.006	0.918
	Rosetta	0.79	0.51	0.54	0.77	---	1.47	1.32	87.32	11.27	1.41	7.35	0.007	1.007
Afp5	Foldit	0.86	0.72	0.75	0.83	0.54	1.13	1.25	95.92	4.08	0	0.69	0.007	0.738
	Microscopist	0.9	0.77	0.81	0.87	0.55	1.45	1.67	90.48	9.52	0	4.83	0.008	0.896
	ARP/wARP	0.88	0.75	0.79	0.85	0.55	2.06	8.38	87.91	10.23	1.86	8.53	0.08	1.493
	Phenix	0.83	0.69	0.73	0.78	0.42	1.95	4.14	78.45	21.55	0	6.73	0.008	1.225
	Buccaneer	0.84	0.70	0.74	0.80	0.50	1.72	4.82	92.41	7.59	0	3.05	0.009	1.167
	Rosetta	0.69	0.56	0.58	0.65	---	1.59	1.66	84.14	15.17	0.69	9.22	0.006	1.004
Afp7	Foldit	0.86	0.72	0.75	0.84	0.52	0.84	1.2	98.13	1.87	0	0.47	0.004	0.667
	Microscopist	0.87	0.75	0.79	0.85	0.50	1.83	4.81	88.79	11.21	0	8.02	0.006	1.082
	ARP/wARP	0.88	0.75	0.79	0.85	0.53	2.06	8.38	87.91	10.23	1.86	8.53	0.008	1.493
	Phenix	0.8	0.69	0.72	0.74	0.45	2.05	6.18	81.82	17.05	1.14	0	0.008	1.392
	Buccaneer	0.85	0.73	0.76	0.82	0.50	1.88	5.98	90.00	10.00	0	5.71	0.007	1.083
	Rosetta	0.66	0.44	0.47	0.61	---	1.6	2.1	87.02	12.98	0	6.5	0.005	0.863
Afp9	Foldit	0.85	0.75	0.78	0.83	0.47	1.06	2.75	98.21	1.79	0	1.82	0.009	0.829
	Microscopist	0.87	0.79	0.82	0.85	0.46	1.58	2.76	91.07	7.14	1.79	6.36	0.007	1.082
	ARP/wARP	0.86	0.78	0.81	0.84	0.47	2.19	10.27	86.11	13.89	0	11.32	0.007	0.952
	Phenix	0.83	0.73	0.76	0.8	0.41	1.68	2.78	87.18	12.82	0	5	0.006	1.042
	Buccaneer	0.81	0.69	0.72	0.78	0.41	2.00	5.21	80.85	17.02	2.13	6.67	0.009	1.251
	Rosetta	0.81	0.72	0.74	0.79	---	1.69	3.85	91.07	8.93	0	4.55	0.007	1.476

For each of the 4 different proteins, any method that outperformed the other 5 for a particular metric is shaded in green, with any method outperformed by the other 5 shaded red. CC_mask_, CC_box_, CC_peaks_, and CC_volume_ are correlation coefficients calculated between the model and the map. The differences between these correlation coefficients arise from whether the entire map is used (CC_box_), only the map around the atomic centers (CC_mask_), the molecular envelope defined by the model (CC_volume_), or the strongest peaks in the model and map (CC_peaks_) [14]. CaBLAM uses the geometry of Cɑ atoms to evaluate low-resolution structures [35]. Clashscore reports on the number and severity of steric clashes in a model, and Molprobity score combines the Clashscore with other geometric factors to provide an overall evaluation of model quality [35].

AFP, antefeeding prophage; FSC, Fourier shell correlation; Rama., Ramachandran; RMSD, root mean square deviation

The Foldit score function appears to correctly reflect model quality. As expected, because of the absence of the phase problem, and unlike previous Foldit collaborations with crystallographic data [25,30], the structures from Foldit players that were the best in each puzzle as determined by Phenix validation [14] were also the best according to the Foldit score function, which is based on the Rosetta score function [31], with terms that model properties such as electrostatics, hydrogen bonds, solvation, and torsion angles, with an additional parameter that accounts for electron potential map fit. This observation suggests that by collaborating with Foldit, only minimal work will be required by microscopists to obtain an accurate, high-quality model.

Building models of large molecules into low-resolution data can be a time-consuming process for microscopists building structures by hand. However, in the 4 datasets presented here, Foldit players had arrived at finished structures in less than 48 hours (S16 Fig). Examining the workflow of Foldit players revealed that different players used distinct strategies in their model building. In the case of Afp9, the winning players chose to prioritize map fitting first and waited until the end to optimize the geometry of the structure (S1 Movie). Alternatively, in the case of Afp5, the winning players instead performed geometry optimization intermittently over the course of map fitting (S2 Movie). The general consensus—among the winning players who generated these 4 Foldit solutions—was to fold the protein “by hand” in the early stages of the puzzle and then run “recipes” (in-game algorithms written by the players) toward the end of the puzzle. Detailed accounts from all of the Foldit players who produced these 4 models are described in the “Foldit Player Testimonials” section in S2 Text.

These results indicate that there are multiple routes toward cryo-EM model building and that Foldit players could greatly speed the arduous model-building process for many cryo-EM projects. Although collaborating with Foldit players currently requires contacting the Foldit developers, future developments will include the ability for cryo-EM researchers the ability to communicate with Foldit players easily.

The strategy described here takes advantage of the collective ability of nonprofessional citizen scientists; however, the Foldit modeling tools are also available for individuals. Foldit Standalone runs offline on a single workstation and can be used by researchers to build and refine their structures with the Foldit scoring function [32]. Alternatively, Foldit Custom Contests can now be administered by researchers to allow online, collaborative model building and refinement among a research group or department or even a class of students [33]. Although we anticipate that for best results, researchers should draw on the collective expertise of the Foldit players, these other options may be attractive in the very competitive cryo-EM field.

To conclude, with the rapid improvement of cryo-EM map quality, it is now paramount for our building and refinement skills and tools to improve commensurably. Enlisting the help of citizen scientists, such as Foldit players, is one option to do so.

## Materials and methods

To generate puzzles for Foldit players, the cryo-EM map (EMD-4782) sharpened with an overall b-factor of 105 Å^2^ [34] was segmented around each fitted monomer of Afp1, Afp5, Afp7, and Afp9 (PDB 6rao, [34]), with a radius of 3 Å around fitted atoms. For a detailed description of the Foldit puzzle setup and order, please see S1 Text. To calculate the FSC between models and map, a single version of an unfiltered, unsharpened segmented map was generated for each target by keeping a zone enclosing all fitted models (Microscopist, Rosetta, Phenix, Foldit) with a radius of 3 Å around fitted atoms. The FSC was then calculated between the segmented map and a simulated map (up to Nyquist resolution with same pixel spacing) from each fitted model.

### Ethics statement

Foldit has received IRB approval, and Foldit players provided informed consent to participate in research (University of Washington IRB STUDY00001238, titled: "Scientific Discovery Games").

## Supporting information

S1 Data Underlying key geometric and map fit parameters for data for Afp7. (XLSX)

S2 Data Underlying key geometric and map fit parameters for data for Afp1. (XLSX)

S3 Data Underlying key geometric and map fit parameters for data for Afp5. (XLSX)

S4 Data Underlying key geometric and map fit parameters for data for Afp9. (XLSX)

S1 Table Cα RMSDs between different models (in Å). Rosetta and Buccaneer models not shown, as they were incomplete.
(DOCX)

S1 Fig Comparison of model building for Afp1 in (A) an overall view, and (B and C) views to compare side-chain fitting. The Foldit structure is rendered in green, the microscopist structure in gray, the Phenix model in magenta, and Rosetta model in yellow. Because of the large deviations from the other structures, the Rosetta model is omitted in the zoomed-in views in parts B and C. Electron potential map is contoured at 2 *σ*. Afp1, antefeeding prophage 1.
(PNG)

S2 Fig Comparison of model building for Afp5 in (A) an overall view, and (B and C) views to compare side-chain fitting. The Foldit structure is rendered in green, the microscopist structure in gray, the Phenix model in magenta, and Rosetta model in yellow. Because of the large deviations from the other structures, the Rosetta model is omitted in the zoomed-in views in parts B and C. Electron potential map is contoured at 2 *σ*. Afp5, antefeeding prophage 5.
(PNG)

S3 Fig Comparison of model building for Afp9 in (A) an overall view, and (B and C) views to compare side- chain fitting. The Foldit structure is rendered in green, the microscopist structure in gray, the Phenix model in magenta, and Rosetta model in yellow. Because of the large deviations from the other structures, the Rosetta model is omitted in the zoomed-in views in parts B and C. Electron potential map is contoured at 2 *σ*. Afp9, antefeeding prophage 9.
(PNG)

S4 Fig Comparison of model building for Afp1 in (A) an overall view, and (B and C) views to compare side-chain fitting. The Foldit structure is rendered in green, ARP/wARP in orange, and Buccaneer in blue. Electron potential map is contoured at 2 *σ*. Afp1, antefeeding prophage 1.
(PNG)

S5 Fig Comparison of model building for Afp5 in (A) an overall view, and (B and C) views to compare side-chain fitting. The Foldit structure is rendered in green, ARP/wARP in orange, and Buccaneer in blue. Electron potential map is contoured at 2 *σ*. Afp5, antefeeding prophage 5.
(PNG)

S6 Fig Comparison of model building for Afp7 in (A) an overall view, and (B and C) views to compare side-chain fitting. The Foldit structure is rendered in green, ARP/wARP in orange, and Buccaneer in blue. Electron potential map is contoured at 2 *σ*. Afp7, antefeeding prophage 7.
(PNG)

S7 Fig Comparison of model building for Afp9 in (A) an overall view, and (B and C) views to compare side-chain fitting. The Foldit structure is rendered in green, ARP/wARP in orange, and Buccaneer in blue. Electron potential map is contoured at 2 *σ*. Afp9, antefeeding prophage 9.
(PNG)

S8 Fig Comparison of key geometric and map fit parameters for each of the tested cases for Afp7. (A) Comparison of Ramachandran outlier and allowed backbone conformations. (B) Comparison of Molprobity Clashscore. (C) Comparison of 3 different map-to-model correlation coefficients. Underlying data for these graphs are provided in S1 Data. Afp7, antefeeding prophage 7.
(PNG)

S9 Fig Comparison of key geometric and map fit parameters for each of the tested cases for Afp1. (A) Comparison of Ramachandran outlier and allowed backbone conformations. (B) Comparison of Molprobity Clashscore. (C) Comparison of 3 different map-to-model correlation coefficients. Underlying data for these graphs are provided in S2 Data. Afp1, antefeeding prophage 1.
(PNG)

S10 Fig Comparison of key geometric and map fit parameters for each of the tested cases for Afp5. (A) Comparison of Ramachandran outlier and allowed backbone conformations. (B) Comparison of Molprobity Clashscore. (C) Comparison of 3 different map-to-model correlation coefficients. Underlying data for these graphs are provided in S3 Data. Afp5, antefeeding prophage 5.
(PNG)

S11 Fig Comparison of key geometric and map fit parameters for each of the tested cases for Afp9. (A) Comparison of Ramachandran outlier and allowed backbone conformations. (B) Comparison of Molprobity Clashscore. (C) Comparison of 3 different map-to-model correlation coefficients. Underlying data for these graphs are provided in S4 Data. Afp9, antefeeding prophage 9.
(PNG)

S12 Fig Map versus model FSC curves for (A) Afp1, (B) Afp5, and (C) Afp9, comparing the Microscopist (gray), Foldit (green), and Phenix (purple) models. In each case, the hand-built models outperformed the Phenix and Buccaneer models, with the microscopist, ARP w/ARP, and Foldit models displaying similar fit. Afp, antefeeding prophage; FSC, Fourier shell correlation.
(PNG)

S13 Fig Rosetta Energy versus GDT_TS plots for Foldit Puzzles 1554 and 1572: CASP13 target T1021s1 (the closer a model is to 1, on the right, the closer it matches the native fold). (A) In Foldit puzzle 1554, players were unable to get close to the native state when only starting from server models without any experimental data. Each green point represents a Foldit player prediction. (B) In Foldit puzzle 1572, however, players were able to reach the native state when provided with a cryo-EM density map. cryo-EM, cryo-electron microscopy; GDT_TS, global distance test.
(PNG)

S14 Fig Rosetta Energy versus GDT_TS plots for Foldit Puzzles 1579 and 1588: CASP13 target T1022s1 (the closer a model is to 1, on the right, the closer it matches the native fold). (A) In Foldit puzzle 1579, players were unable to get close to the native state when only starting from server models without any experimental data. (B) In Foldit puzzle 1588, however, players were able to reach the native state when provided with a cryo-EM density map. cryo-EM, cryo-electron microscopy; GDT_TS, global distance test.
(PNG)

S15 Fig Screenshot of starting state for Foldit Puzzle 1598. Players were only given an extended chain along with the cryo-EM density map. cryo-EM, cryo-electron microscopy.
(PNG)

S16 Fig Rosetta Energy versus GDT_TS plot of Foldit Puzzle 1598 (the closer a model is to 1, on the right, the closer it matches the native fold). Starting from an extended chain, showing the progression of play over the first 2 days of the puzzle. Although no one was able to reach the native state in the first 24 hours (A), the native topology was found by the second day (B). GDT_TS, global distance test.
(PNG)

S17 Fig Rosetta Energy versus GDT_TS plot of Foldit Puzzle 1598 after the puzzle ended (the closer a model is to 1, on the right, the closer it matches the native fold). Final plot, after the puzzle closed, of the GDT_TS score versus the Rosetta Energy. GDT_TS, global distance test.
(PNG)

S18 Fig Tracking Foldit player actions during Puzzle 1588: (A) Comments on shared player solutions. (B) Recipe additions to Notes for various segments.
(PNG)

S19 Fig Poor player results from the first round (Puzzle 1579) without density as a guide. Source: Foldit blog 10/16/18 https://fold.it/portal/node/2006086). A value of 1 represents a perfect match with the native.
(PNG)

S20 Fig Starting by matching a tryptophan (“8” shape, top left) and related helix, and a phenylalanine (“9” shape, center) and related sheet. The rest of the protein is cut out for visibility (bottom right).
(PNG)

S21 Fig Hand-folding 1 hour and refining with recipes 24 hours almost every day. (PNG)

S22 Fig Two extra winning solutions shared to the group after the deadline by *jeff101*. (A) The latest “B2p8” solution. (B) Latest “Batz” solution shared by player *jeff101*.
(PNG)

S1 Text Supplemental Results. (DOCX)

S2 Text Foldit Player Testimonials from all 6 players who contributed to the 4 Foldit models. (DOCX)

S1 Authors Membership list of the Foldit Players consortium. (DOCX)

S1 Movie Winning players for Afp9 prioritized map fitting first over geometry optimization. (MP4)

S2 Movie Winning players for Afp5 performed geometry optimization intermittently during the puzzle. (MOV)

## Abbreviations

AFP: antefeeding prophage
cryo-EM: cryo-electron microscopy
FSC: Fourier shell correlation
RMSD: root mean square deviation
Rama: Ramachandran

## References

[1] Baker M. Cryo-electron microscopy shapes up. Nature. 2018;561(7724):565–7. Epub 2018/09/27. 10.1038/d41586-018-06791-6 . 30254359

[2] Binshtein E, Ohi MD. Cryo-electron microscopy and the amazing race to atomic resolution. Biochemistry. 2015;54(20):3133–41. Epub 2015/05/09. 10.1021/acs.biochem.5b00114 . 25955078

[3] Saibil HR. Blob-ology and biology of cryo-EM: an interview with Helen Saibil. BMC Biol. 2017;15(1):77 Epub 2017/09/02. 10.1186/s12915-017-0417-z 28859647 PMC5580197

[4] Smith MT, Rubinstein JL. Structural biology. Beyond blob-ology. Science. 2014;345(6197):617–9. Epub 2014/08/12. 10.1126/science.1256358 . 25104368

[5] Gonen T, Cheng Y, Sliz P, Hiroaki Y, Fujiyoshi Y, Harrison SC, et al Lipid-protein interactions in double-layered two-dimensional AQP0 crystals. Nature. 2005;438(7068):633–8. Epub 2005/12/02. 10.1038/nature04321 16319884 PMC1350984

[6] Henderson R, Baldwin JM, Ceska TA, Zemlin F, Beckmann E, Downing KH. Model for the structure of bacteriorhodopsin based on high-resolution electron cryo-microscopy. J Mol Biol. 1990;213(4):899–929. Epub 1990/06/20. 10.1016/S0022-2836(05)80271-2 . 2359127

[7] Kuhlbrandt W, Wang DN, Fujiyoshi Y. Atomic model of plant light-harvesting complex by electron crystallography. Nature. 1994;367(6464):614–21. Epub 1994/02/17. 10.1038/367614a0 . 8107845

[8] Nogales E, Wolf SG, Downing KH. Structure of the alpha beta tubulin dimer by electron crystallography. Nature. 1998;391(6663):199–203. Epub 1998/01/15. 10.1038/34465 . 9428769

[9] Unwin N. Acetylcholine receptor channel imaged in the open state. Nature. 1995;373(6509):37–43. Epub 1995/01/05. 10.1038/373037a0 . 7800037

[10] Yu X, Jin L, Zhou ZH. 3.88 A structure of cytoplasmic polyhedrosis virus by cryo-electron microscopy. Nature. 2008;453(7193):415–9. Epub 2008/05/02. 10.1038/nature06893 18449192 PMC2746981

[11] Zhang X, Settembre E, Xu C, Dormitzer PR, Bellamy R, Harrison SC, et al Near-atomic resolution using electron cryomicroscopy and single-particle reconstruction. Proc Natl Acad Sci U S A. 2008;105(6):1867–72. Epub 2008/02/02. 10.1073/pnas.0711623105 18238898 PMC2542862

[12] Callaway E. The revolution will not be crystallized: a new method sweeps through structural biology. Nature News. 2015;525(7568):172. 10.1038/525172a 26354465

[13] Wlodawer A, Li M, Dauter Z. High-Resolution Cryo-EM Maps and Models: A Crystallographer's Perspective. Structure. 2017;25(10):1589–97 e1. Epub 2017/09/05. 10.1016/j.str.2017.07.012 28867613 PMC5657611

[14] Afonine PV, Klaholz BP, Moriarty NW, Poon BK, Sobolev OV, Terwilliger TC, et al New tools for the analysis and validation of cryo-EM maps and atomic models. Acta Crystallogr D Struct Biol. 2018;74(Pt 9):814–40. Epub 2018/09/11. 10.1107/S2059798318009324 30198894 PMC6130467

[15] Lawson CL, Chiu W. Comparing cryo-EM structures. J Struct Biol. 2018;204(3):523–6. Epub 2018/10/16. 10.1016/j.jsb.2018.10.004 30321594 PMC6464812

[16] Wang RY, Song Y, Barad BA, Cheng Y, Fraser JS, DiMaio F. Automated structure refinement of macromolecular assemblies from cryo-EM maps using Rosetta. Elife. 2016;5 Epub 2016/09/27. 10.7554/eLife.17219 27669148 PMC5115868

[17] Terwilliger TC, Adams PD, Afonine PV, Sobolev OV. A fully automatic method yielding initial models from high-resolution cryo-electron microscopy maps. Nat Methods. 2018;15(11):905–8. Epub 2018/11/01. 10.1038/s41592-018-0173-1 30377346 PMC6214191

[18] Cowtan K. The Buccaneer software for automated model building. 1. Tracing protein chains. Acta Crystallogr D Biol Crystallogr. 2006;62(Pt 9):1002–11. Epub 2006/08/25. 10.1107/S0907444906022116 . 16929101

[19] Langer G, Cohen SX, Lamzin VS, Perrakis A. Automated macromolecular model building for X-ray crystallography using ARP/wARP version 7. Nat Protoc. 2008;3(7):1171–9. Epub 2008/07/05. 10.1038/nprot.2008.91 18600222 PMC2582149

[20] Lee J, Kladwang W, Lee M, Cantu D, Azizyan M, Kim H, et al RNA design rules from a massive open laboratory. Proc Natl Acad Sci U S A. 2014;111(6):2122–7. Epub 2014/01/29. 10.1073/pnas.1313039111 24469816 PMC3926058

[21] Kim JS, Greene MJ, Zlateski A, Lee K, Richardson M, Turaga SC, et al Space-time wiring specificity supports direction selectivity in the retina. Nature. 2014;509(7500):331–6. Epub 2014/05/09. 10.1038/nature13240 24805243 PMC4074887

[22] Kawrykow A, Roumanis G, Kam A, Kwak D, Leung C, Wu C, Zarour E, Sarmenta L, Blanchette M, and Waldispühl J. Phylo: a citizen science approach for improving multiple sequence alignment. PLoS ONE. 2012: 7(3). e31362 10.1371/journal.pone.0031362 22412834 PMC3296692

[23] Sorensen JJ, Pedersen MK, Munch M, Haikka P, Jensen JH, Planke T, et al Exploring the quantum speed limit with computer games. Nature. 2016;532(7598):210–3. Epub 2016/04/15. 10.1038/nature17620 . 27075097

[24] Cooper S, Khatib F, Treuille A, Barbero J, Lee J, Beenen M, et al Predicting protein structures with a multiplayer online game. Nature. 2010;466(7307):756–60. 10.1038/nature09304 WOS:000280562500039. 20686574 PMC2956414

[25] Horowitz S, Koepnick B, Martin R, Tymieniecki A, Winburn AA, Cooper S, et al Determining crystal structures through crowdsourcing and coursework. Nat Commun. 2016;7:12549 Epub 2016/09/17. 10.1038/ncomms12549 27633552 PMC5028414

[26] Rohl CA, Strauss CE, Misura KM, Baker D. Protein structure prediction using Rosetta. Methods in enzymology. 2004; 383:66–93. 10.1016/S0076-6879(04)83004-0 15063647

[27] DiMaio F, Song Y, Li X, Brunner MJ, Xu C, Conticello V, et al Atomic-accuracy models from 4.5-A cryo-electron microscopy data with density-guided iterative local refinement. Nat Methods. 2015;12(4):361–5. Epub 2015/02/24. 10.1038/nmeth.3286 25707030 PMC4382417

[28] Emsley P, Lohkamp B, Scott WG, Cowtan K. Features and development of Coot. Acta Crystallogr D Biol Crystallogr. 2010;66(Pt 4):486–501. Epub 2010/04/13. 10.1107/S0907444910007493 20383002 PMC2852313

[29] Adams PD, Afonine PV, Bunkoczi G, Chen VB, Davis IW, Echols N, et al PHENIX: a comprehensive Python-based system for macromolecular structure solution. Acta Crystallogr D Biol Crystallogr. 2010;66(Pt 2):213–21. Epub 2010/02/04. 10.1107/S0907444909052925 20124702 PMC2815670

[30] Khatib F, DiMaio F, Foldit Contenders G, Foldit Void Crushers G, Cooper S, Kazmierczyk M, et al Crystal structure of a monomeric retroviral protease solved by protein folding game players. Nat Struct Mol Biol. 2011;18(10):1175–7. Epub 2011/09/20. 10.1038/nsmb.2119 21926992 PMC3705907

[31] Alford RF, Leaver-Fay A, Jeliazkov JR, O'Meara MJ, DiMaio FP, Park H, et al The Rosetta All-Atom Energy Function for Macromolecular Modeling and Design. J Chem Theory Comput. 2017;13(6):3031–48. 10.1021/acs.jctc.7b00125 WOS:000403530100060. 28430426 PMC5717763

[32] Kleffner R, Flatten J, Leaver-Fay A, Baker D, Siegel JB, Khatib F, et al Foldit Standalone: a video game-derived protein structure manipulation interface using Rosetta. Bioinformatics. 2017;33(17):2765–7. Epub 2017/05/10. 10.1093/bioinformatics/btx283 28481970 PMC5860063

[33] Dsilva L, Mittal S, Koepnick B, Flatten J, Cooper S, Horowitz S. Creating custom Foldit puzzles for teaching biochemistry. Biochem Mol Biol Educ. 2019;47(2):133–9. Epub 2019/01/15. 10.1002/bmb.21208 30638297 PMC6428574

[34] Desfosses A, Venugopal H, Joshi T, Felix J, Jessop M, Jeong H, Hyun J, Heymann JB, Hurst MRH, Gutsche I, Mitra AK. Atomic structures of an entire contractile injection system in both the extended and contracted states. Nat Microbiol. 2019 8 5 10.1038/s41564-019-0530-6 31384001 PMC6817355

[35] Williams CJ, Headd JJ, Moriarty NW, Prisant MG, Videau LL, Deis LN, et al MolProbity: More and better reference data for improved all-atom structure validation. Protein Sci. 2018;27(1):293–315. Epub 2017/10/27. 10.1002/pro.3330 29067766 PMC5734394

