# Supplementary material for: Building de novo cryo-electron microscopy structures collaboratively with citizen scientists
Source: PLoS Biol. 2019 Nov 12;17(11):e3000472. doi: 10.1371/journal.pbio.3000472 (PMC6850521; doi:10.1371/journal.pbio.3000472)
Supplement: S1 Table — Rosetta and Buccaneer models not shown, as they were incomplete. (DOCX) [file pbio.3000472.s005.docx]

| **Afp1** | Foldit | Microscopist |
| --- | --- | --- |
| Microscopist | 0.28 | x |
| Phenix | 0.39 | 0.33 |
| ARP/wARP | 0.27 | 0.20 |
| **Afp5** | Foldit | Microscopist |
| Microscopist | 0.31 | x |
| Phenix | 0.42 | 0.36 |
| ARP/wARP | 0.30 | 0.22 |
| **Afp7** | Foldit | Microscopist |
| Microscopist | 0.41 | x |
| Phenix | 0.35 | 0.37 |
| ARP/wARP | 0.33 | 0.38 |
| **Afp9** | Foldit | Microscopist |
| Microscopist | 0.40 | x |
| Phenix | 0.43 | 0.33 |
| ARP/wARP | 0.43 | 0.38 |
