# Supplementary figures and images for: Building de novo cryo-electron microscopy structures collaboratively with citizen scientists
Source: PLoS Biol. 2019 Nov 12;17(11):e3000472. doi: 10.1371/journal.pbio.3000472 (PMC6850521; doi:10.1371/journal.pbio.3000472)

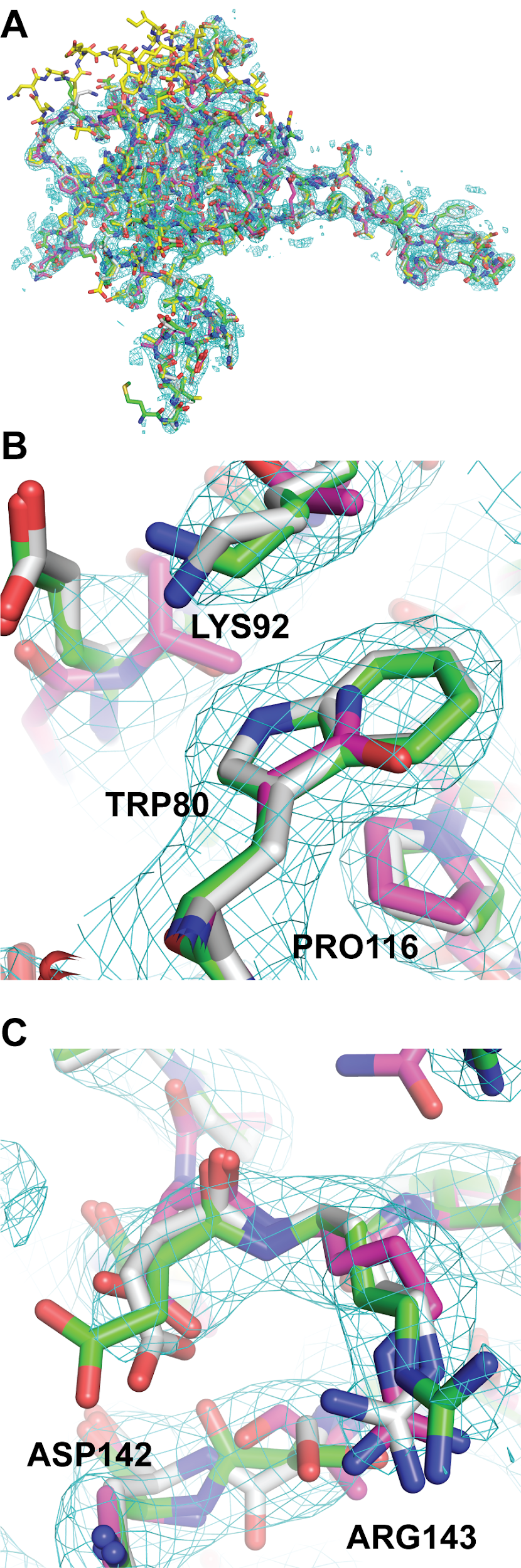

Supplement: S1 Fig — Comparison of model building for Afp1 in (A) an overall view, and (B and C) views to compare side-chain fitting. The Foldit structure is rendered in green, the microscopist structure in gray, the Phenix model in magenta, and Rosetta model in yellow. Because of the large deviations from the other structures, the Rosetta model is omitted in the zoomed-in views in parts B and C. Electron potential map is contoured at 2 σ. Afp1, antefeeding prophage 1. (PNG) [file pbio.3000472.s006.png]

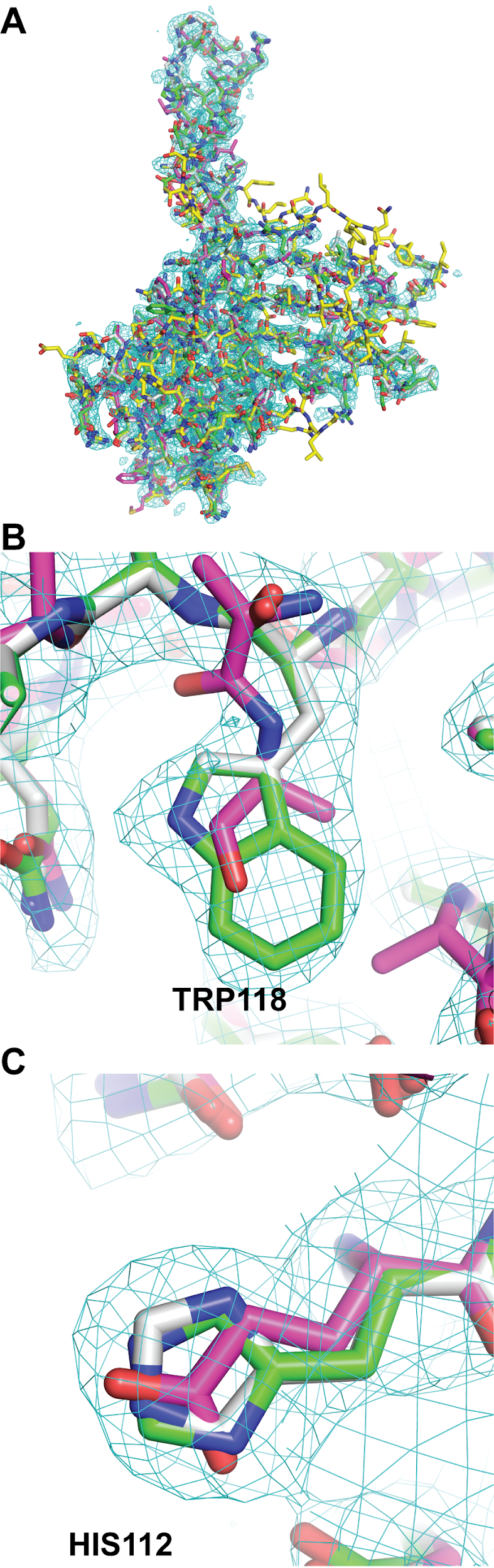

Supplement: S2 Fig — Comparison of model building for Afp5 in (A) an overall view, and (B and C) views to compare side-chain fitting. The Foldit structure is rendered in green, the microscopist structure in gray, the Phenix model in magenta, and Rosetta model in yellow. Because of the large deviations from the other structures, the Rosetta model is omitted in the zoomed-in views in parts B and C. Electron potential map is contoured at 2 σ. Afp5, antefeeding prophage 5. (PNG) [file pbio.3000472.s007.png]

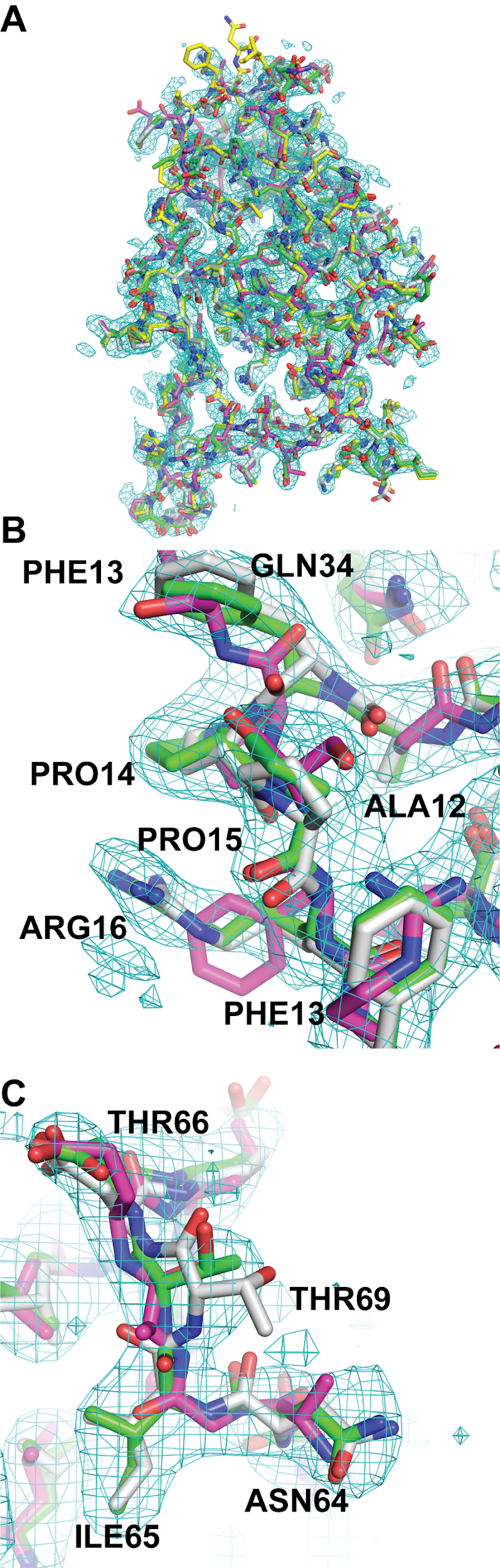

Supplement: S3 Fig — Comparison of model building for Afp9 in (A) an overall view, and (B and C) views to compare side- chain fitting. The Foldit structure is rendered in green, the microscopist structure in gray, the Phenix model in magenta, and Rosetta model in yellow. Because of the large deviations from the other structures, the Rosetta model is omitted in the zoomed-in views in parts B and C. Electron potential map is contoured at 2 σ. Afp9, antefeeding prophage 9. (PNG) [file pbio.3000472.s008.png]

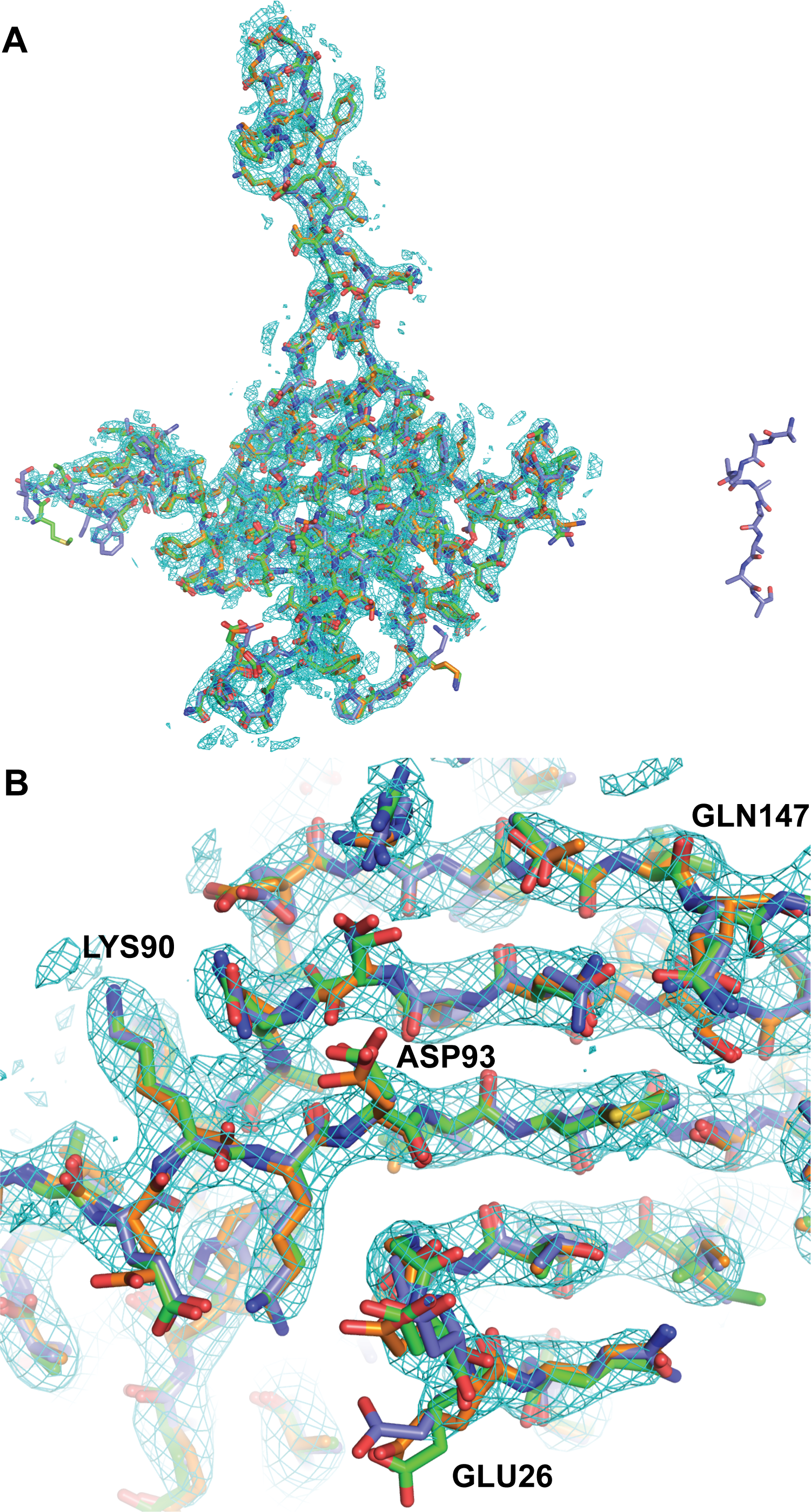

Supplement: S4 Fig — Comparison of model building for Afp1 in (A) an overall view, and (B and C) views to compare side-chain fitting. The Foldit structure is rendered in green, ARP/wARP in orange, and Buccaneer in blue. Electron potential map is contoured at 2 σ. Afp1, antefeeding prophage 1. (PNG) [file pbio.3000472.s009.png]

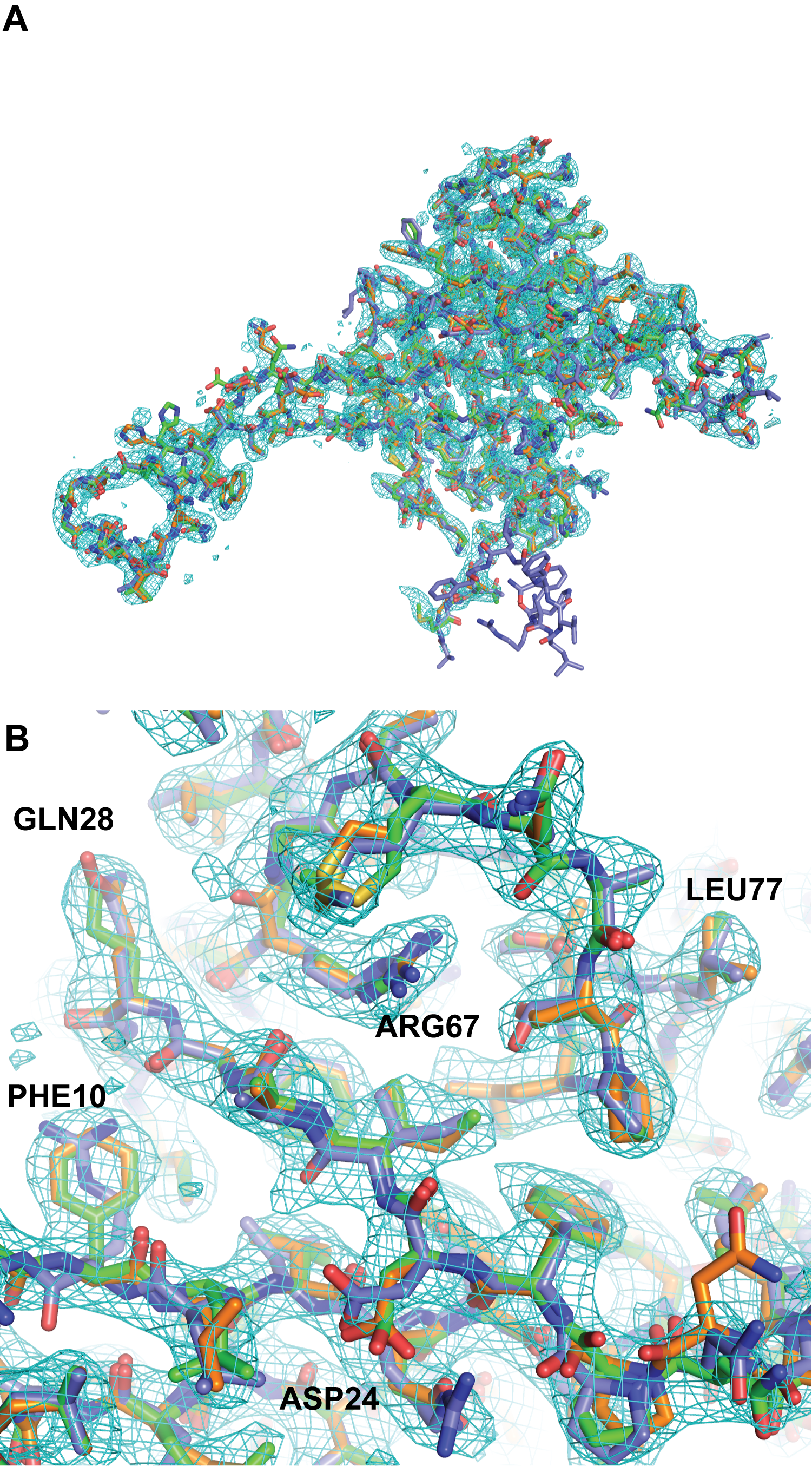

Supplement: S5 Fig — Comparison of model building for Afp5 in (A) an overall view, and (B and C) views to compare side-chain fitting. The Foldit structure is rendered in green, ARP/wARP in orange, and Buccaneer in blue. Electron potential map is contoured at 2 σ. Afp5, antefeeding prophage 5. (PNG) [file pbio.3000472.s010.png]

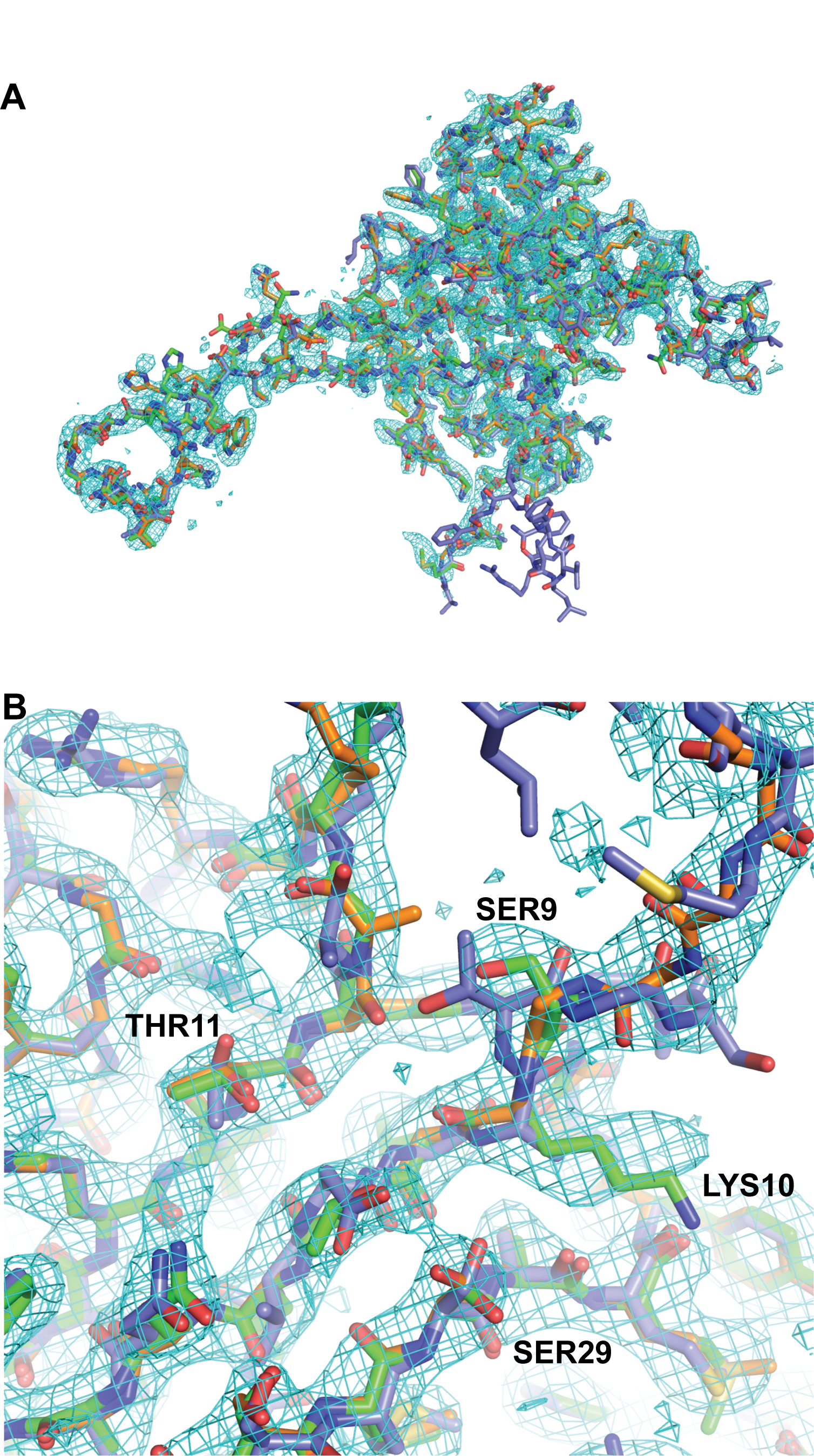

Supplement: S6 Fig — Comparison of model building for Afp7 in (A) an overall view, and (B and C) views to compare side-chain fitting. The Foldit structure is rendered in green, ARP/wARP in orange, and Buccaneer in blue. Electron potential map is contoured at 2 σ. Afp7, antefeeding prophage 7. (PNG) [file pbio.3000472.s011.png]

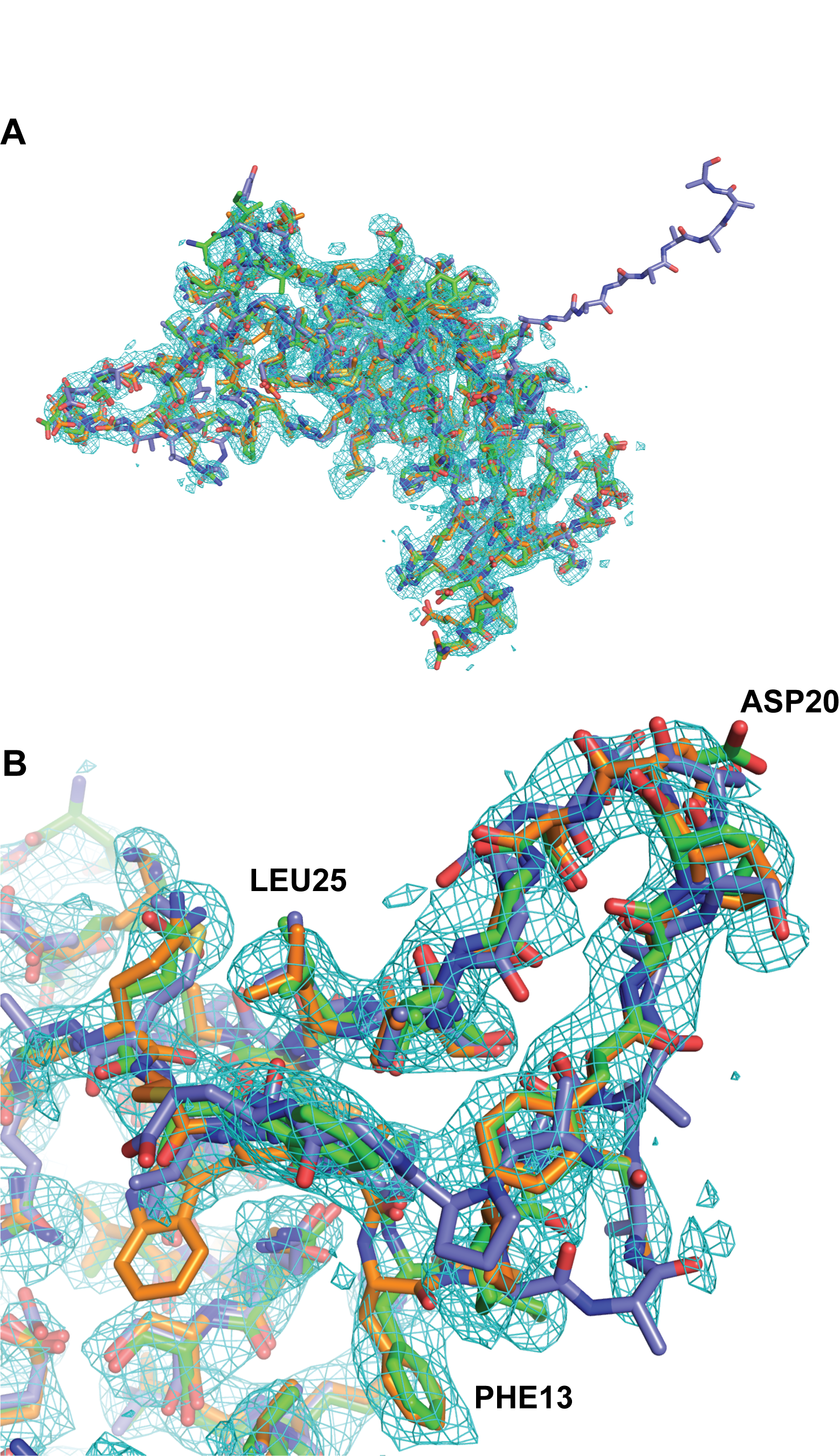

Supplement: S7 Fig — Comparison of model building for Afp9 in (A) an overall view, and (B and C) views to compare side-chain fitting. The Foldit structure is rendered in green, ARP/wARP in orange, and Buccaneer in blue. Electron potential map is contoured at 2 σ. Afp9, antefeeding prophage 9. (PNG) [file pbio.3000472.s012.png]

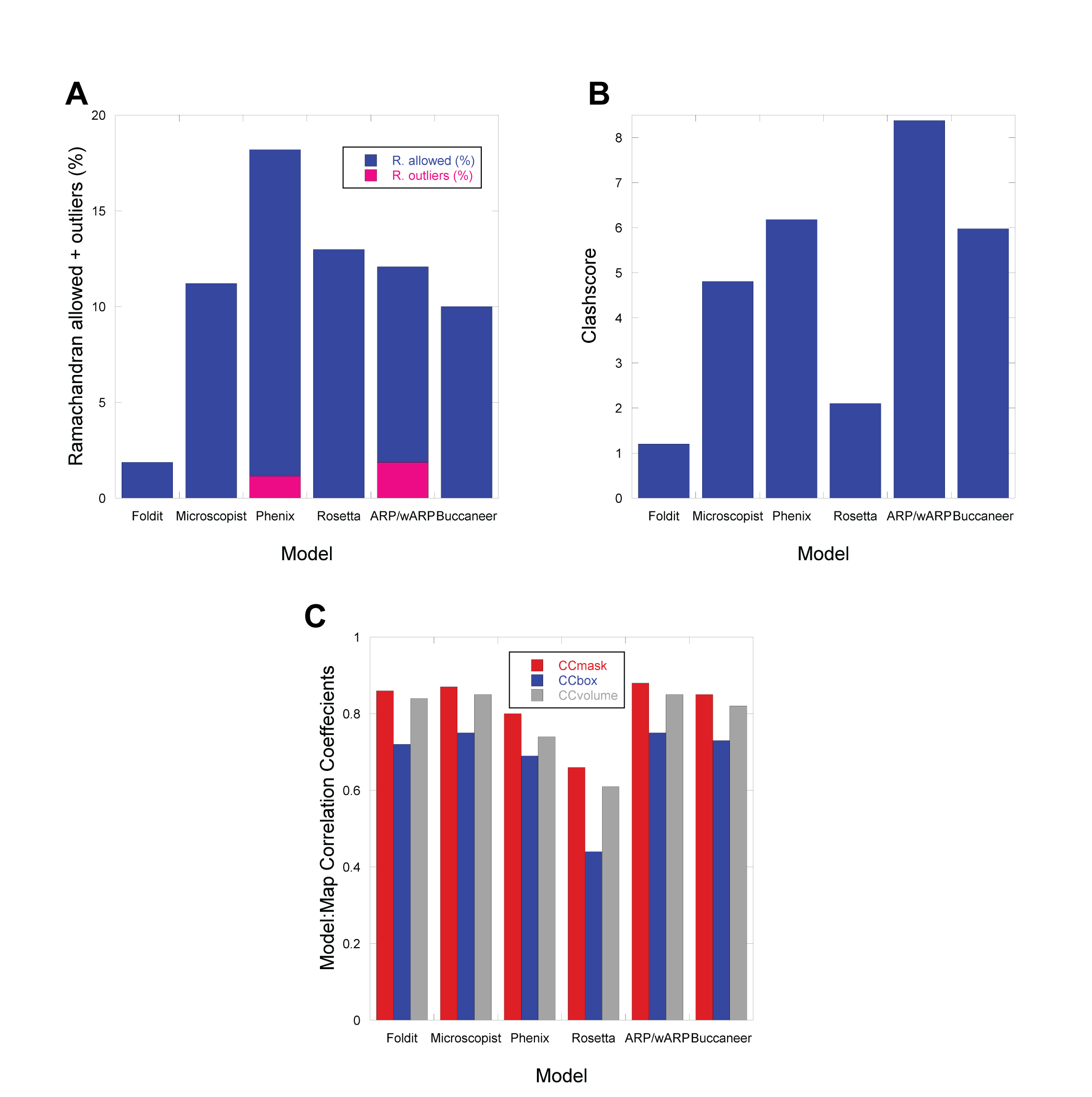

Supplement: S8 Fig — (A) Comparison of Ramachandran outlier and allowed backbone conformations. (B) Comparison of Molprobity Clashscore. (C) Comparison of 3 different map-to-model correlation coefficients. Underlying data for these graphs are provided in S1 Data. Afp7, antefeeding prophage 7. (PNG) [file pbio.3000472.s013.png]

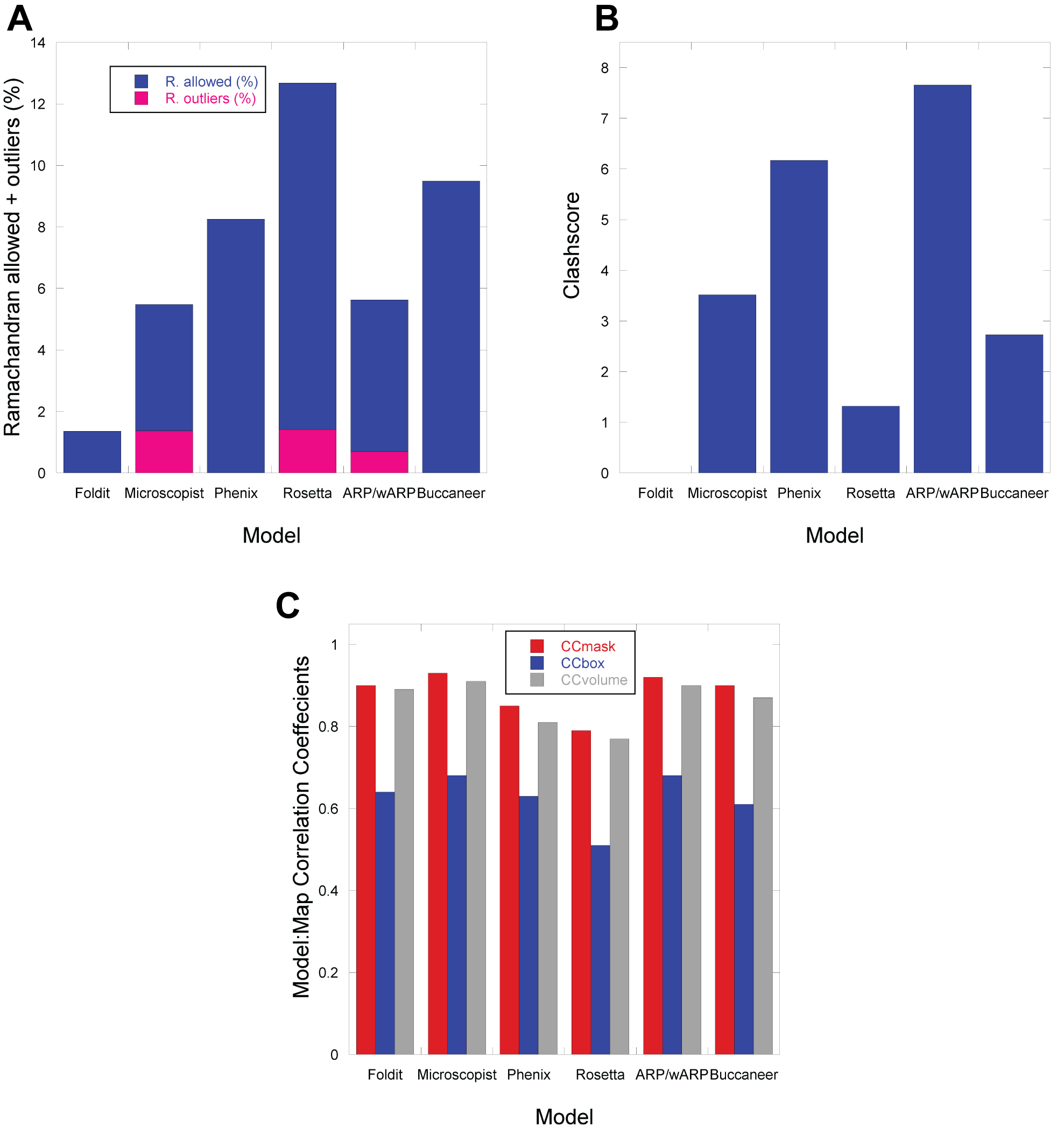

Supplement: S9 Fig — (A) Comparison of Ramachandran outlier and allowed backbone conformations. (B) Comparison of Molprobity Clashscore. (C) Comparison of 3 different map-to-model correlation coefficients. Underlying data for these graphs are provided in S2 Data. Afp1, antefeeding prophage 1. (PNG) [file pbio.3000472.s014.png]

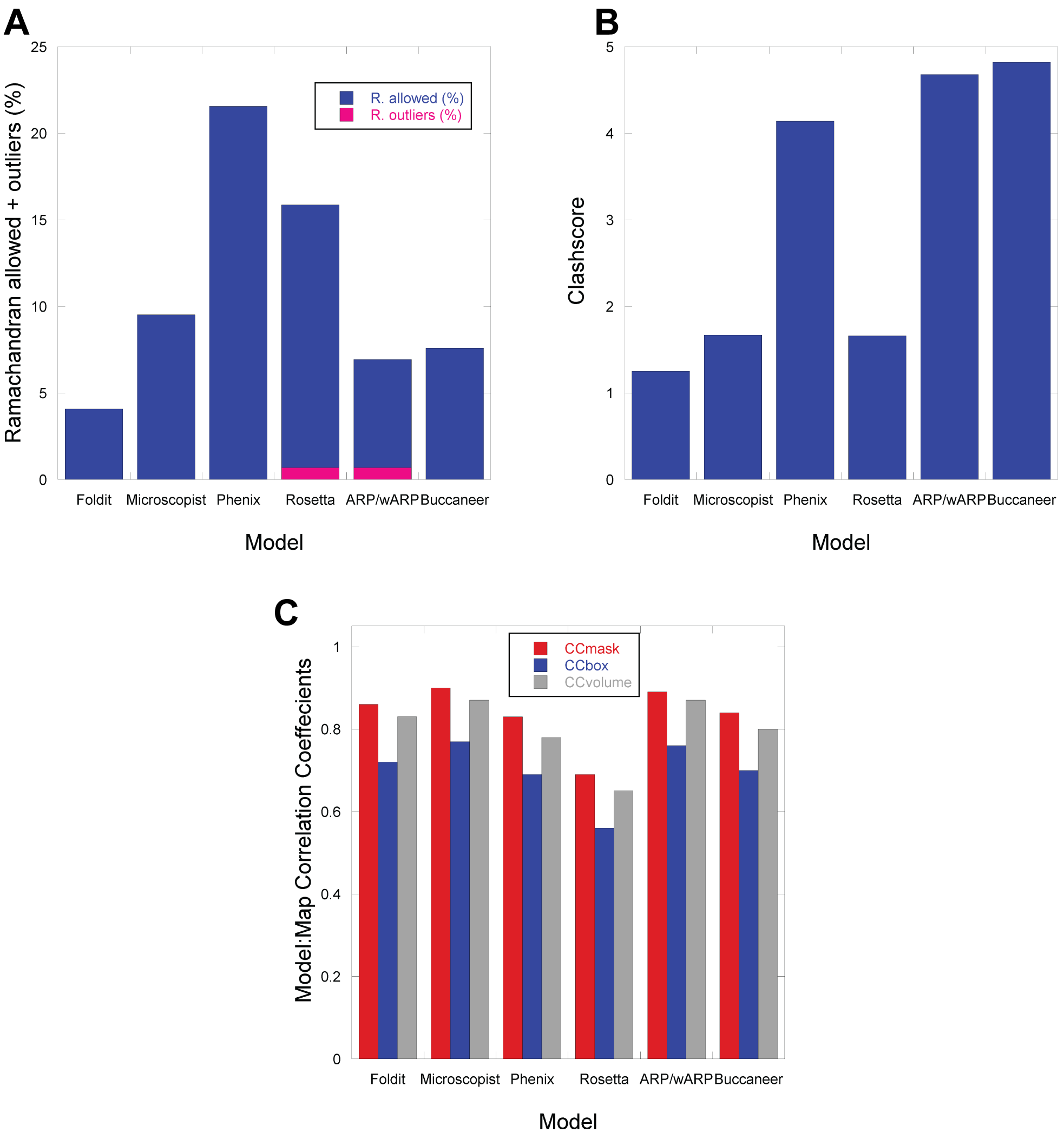

Supplement: S10 Fig — (A) Comparison of Ramachandran outlier and allowed backbone conformations. (B) Comparison of Molprobity Clashscore. (C) Comparison of 3 different map-to-model correlation coefficients. Underlying data for these graphs are provided in S3 Data. Afp5, antefeeding prophage 5. (PNG) [file pbio.3000472.s015.png]

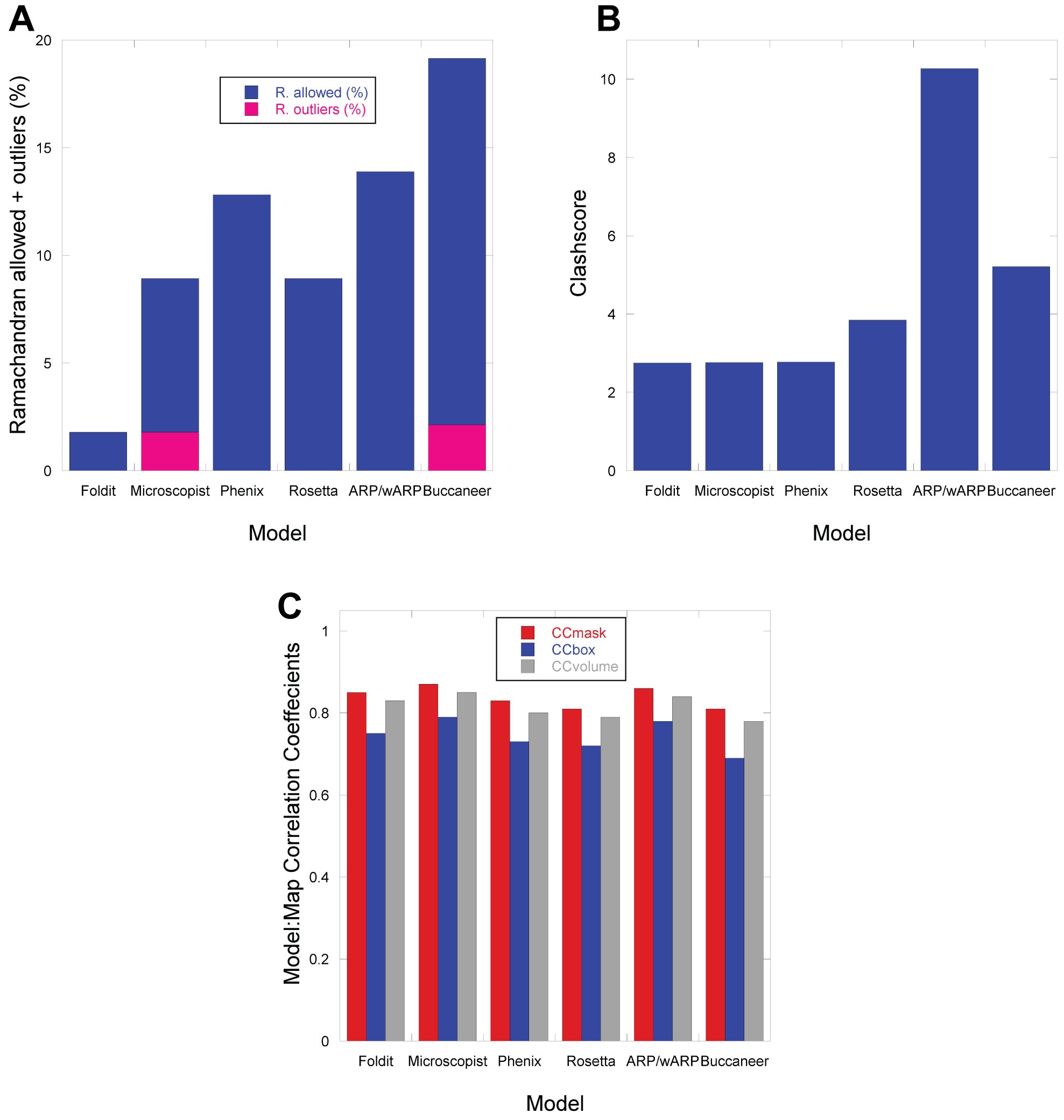

Supplement: S11 Fig — (A) Comparison of Ramachandran outlier and allowed backbone conformations. (B) Comparison of Molprobity Clashscore. (C) Comparison of 3 different map-to-model correlation coefficients. Underlying data for these graphs are provided in S4 Data. Afp9, antefeeding prophage 9. (PNG) [file pbio.3000472.s016.png]

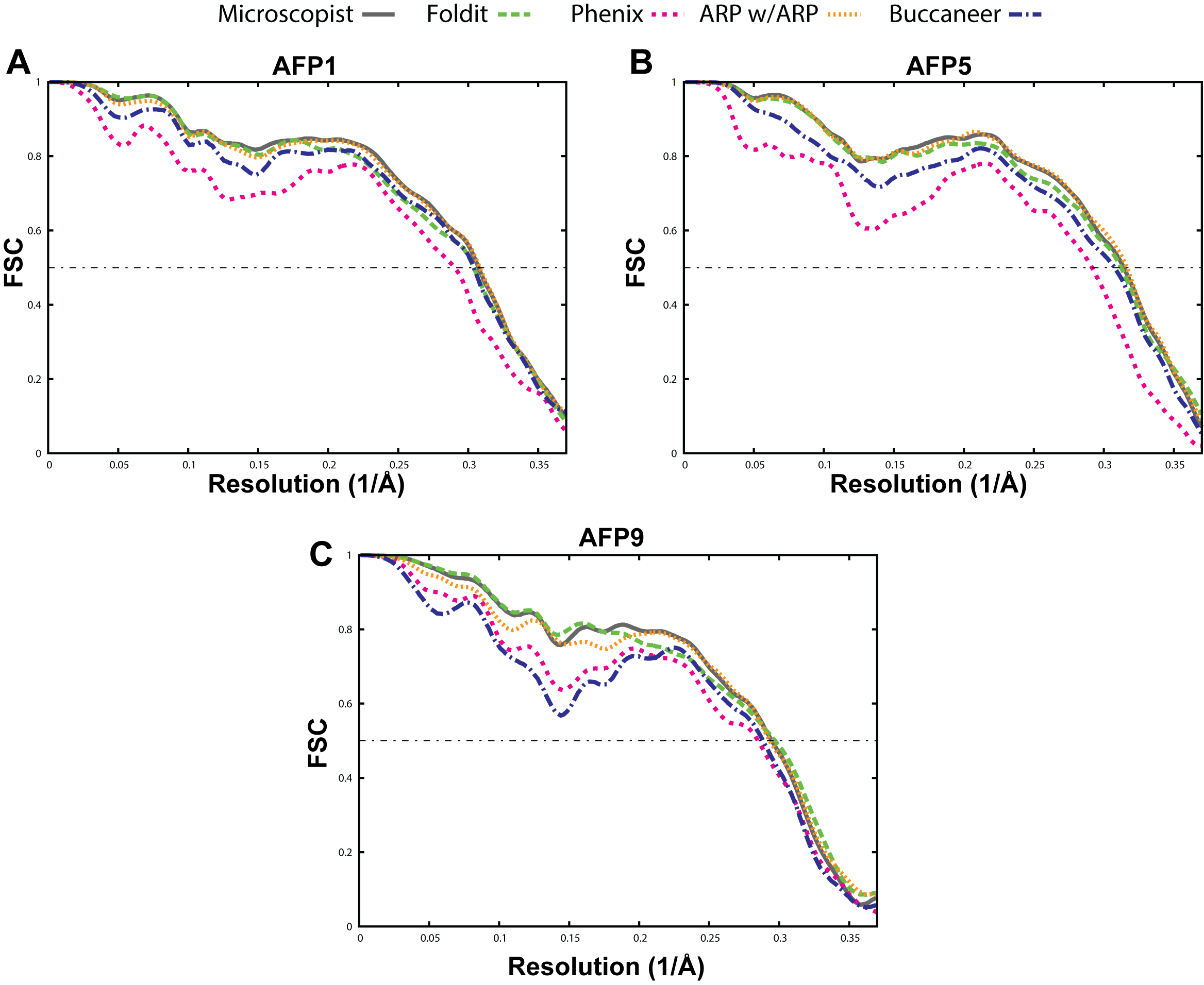

Supplement: S12 Fig — Map versus model FSC curves for (A) Afp1, (B) Afp5, and (C) Afp9, comparing the Microscopist (gray), Foldit (green), and Phenix (purple) models. In each case, the hand-built models outperformed the Phenix and Buccaneer models, with the microscopist, ARP w/ARP, and Foldit models displaying similar fit. Afp, antefeeding prophage; FSC, Fourier shell correlation. (PNG) [file pbio.3000472.s017.png]

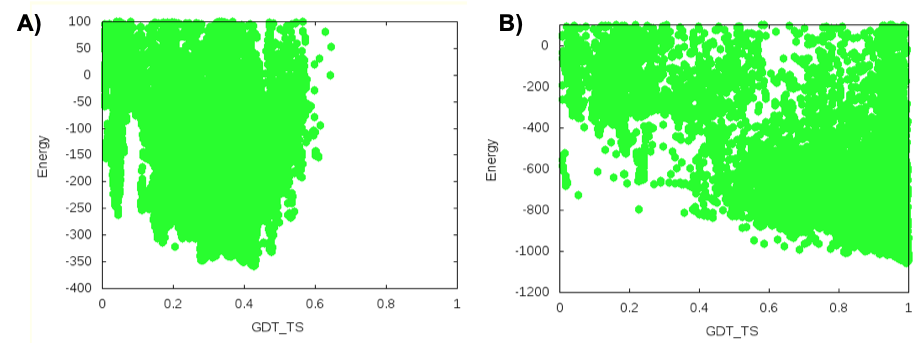

Supplement: S13 Fig — (A) In Foldit puzzle 1554, players were unable to get close to the native state when only starting from server models without any experimental data. Each green point represents a Foldit player prediction. (B) In Foldit puzzle 1572, however, players were able to reach the native state when provided with a cryo-EM density map. cryo-EM, cryo-electron microscopy; GDT_TS, global distance test. (PNG) [file pbio.3000472.s018.png]

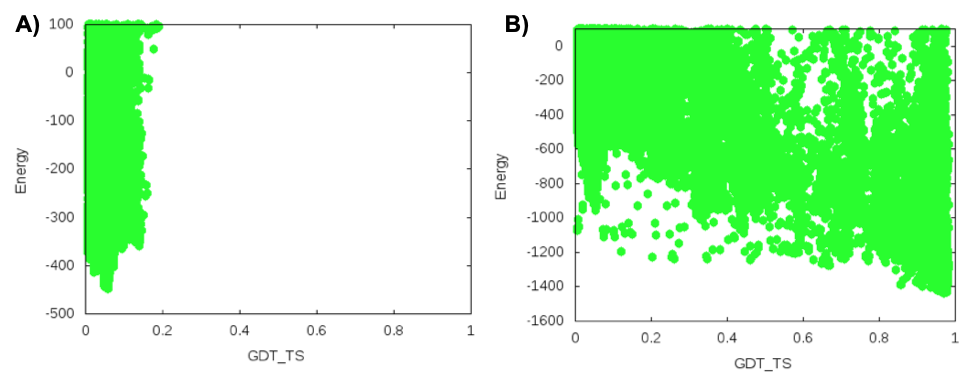

Supplement: S14 Fig — (A) In Foldit puzzle 1579, players were unable to get close to the native state when only starting from server models without any experimental data. (B) In Foldit puzzle 1588, however, players were able to reach the native state when provided with a cryo-EM density map. cryo-EM, cryo-electron microscopy; GDT_TS, global distance test. (PNG) [file pbio.3000472.s019.png]

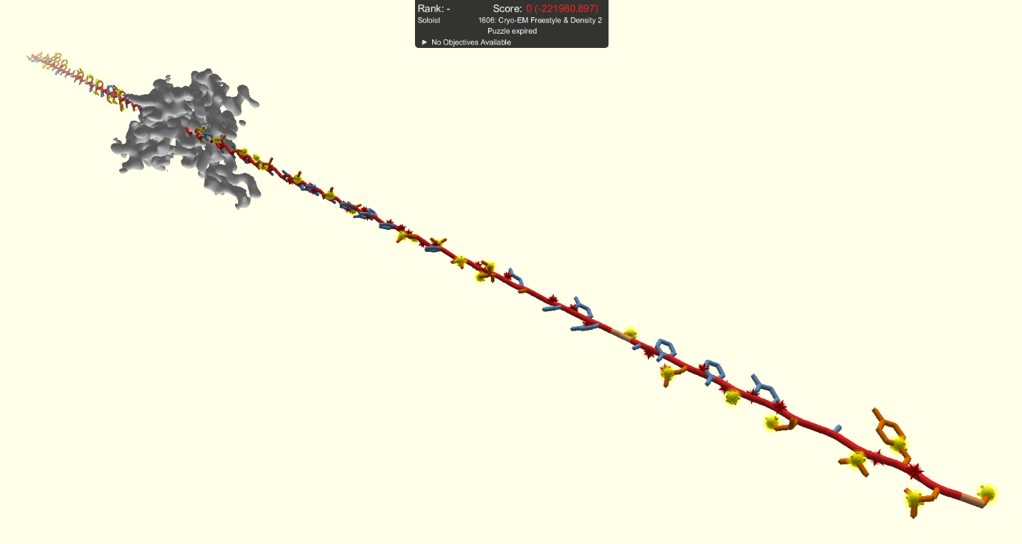

Supplement: S15 Fig — Players were only given an extended chain along with the cryo-EM density map. cryo-EM, cryo-electron microscopy. (PNG) [file pbio.3000472.s020.png]

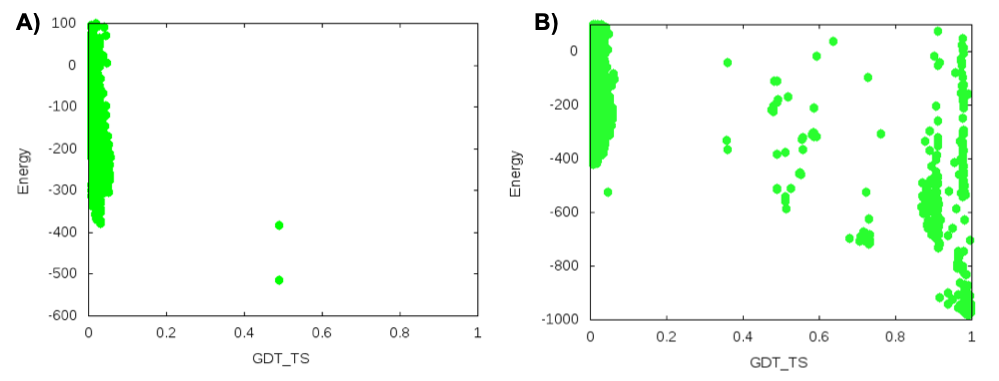

Supplement: S16 Fig — Starting from an extended chain, showing the progression of play over the first 2 days of the puzzle. Although no one was able to reach the native state in the first 24 hours (A), the native topology was found by the second day (B). GDT_TS, global distance test. (PNG) [file pbio.3000472.s021.png]

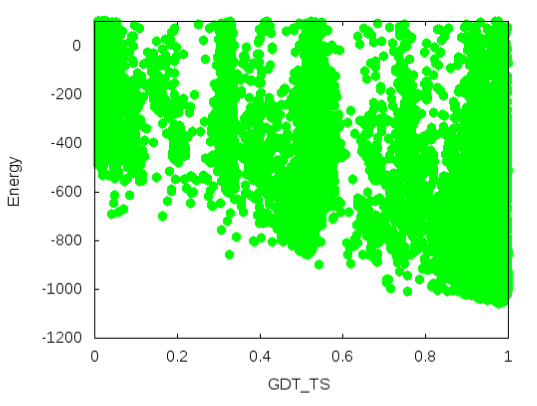

Supplement: S17 Fig — Final plot, after the puzzle closed, of the GDT_TS score versus the Rosetta Energy. GDT_TS, global distance test. (PNG) [file pbio.3000472.s022.png]

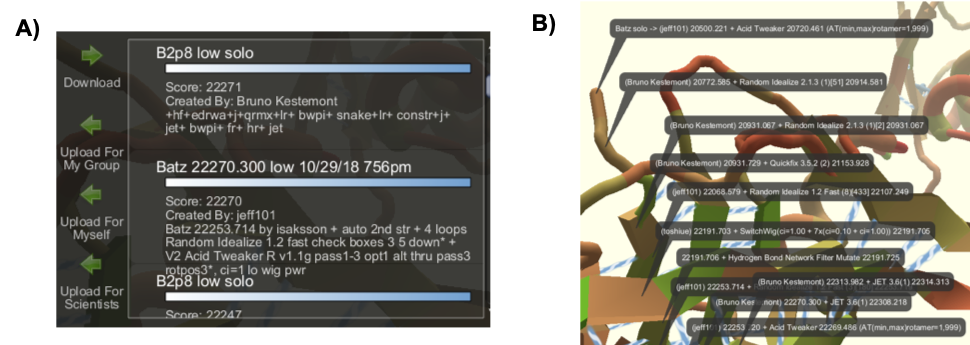

Supplement: S18 Fig — Tracking Foldit player actions during Puzzle 1588: (A) Comments on shared player solutions. (B) Recipe additions to Notes for various segments. (PNG) [file pbio.3000472.s023.png]

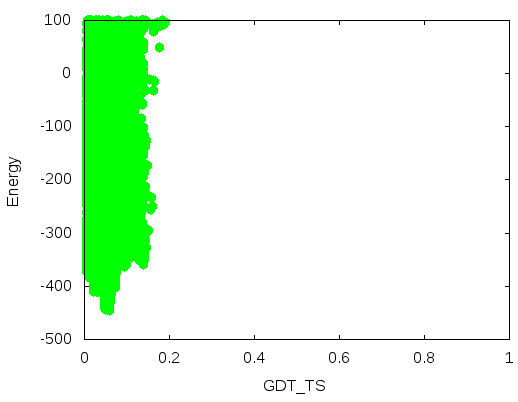

Supplement: S19 Fig — Source: Foldit blog 10/16/18 https://fold.it/portal/node/2006086). A value of 1 represents a perfect match with the native. (PNG) [file pbio.3000472.s024.png]

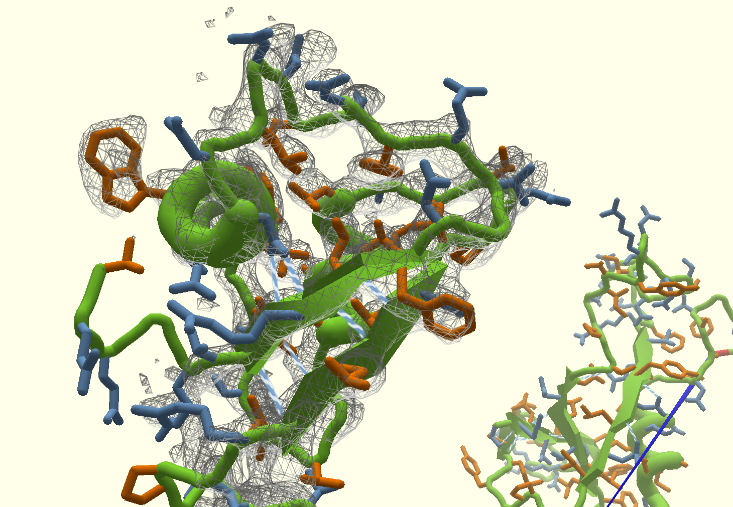

Supplement: S20 Fig — The rest of the protein is cut out for visibility (bottom right). (PNG) [file pbio.3000472.s025.png]

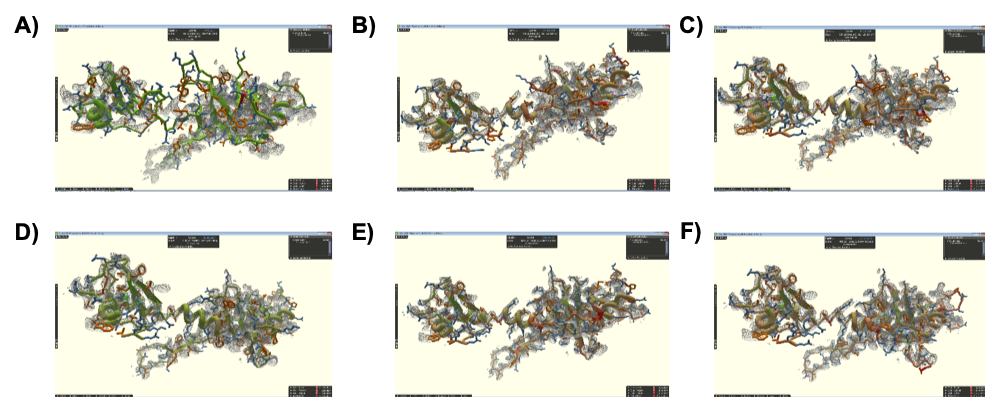

Supplement: S21 Fig — (PNG) [file pbio.3000472.s026.png]

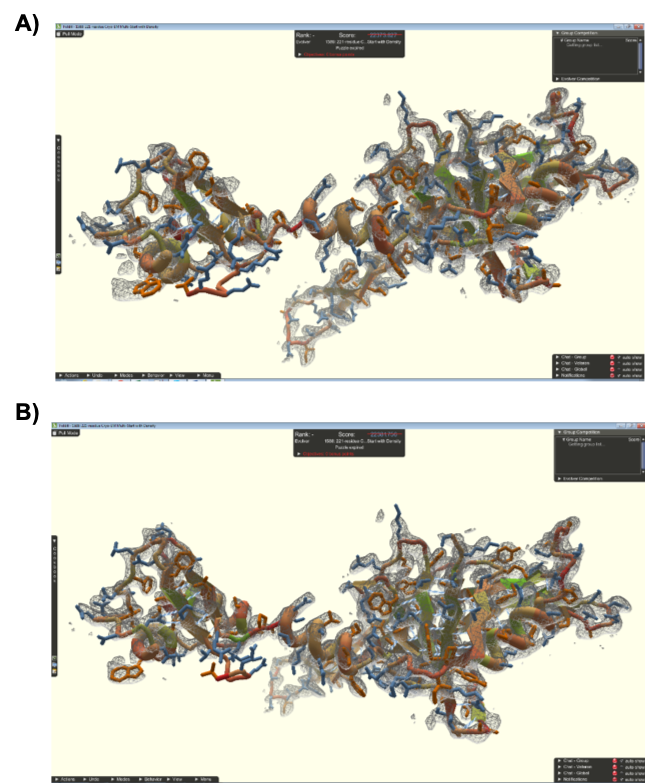

Supplement: S22 Fig — (A) The latest “B2p8” solution. (B) Latest “Batz” solution shared by player jeff101. (PNG) [file pbio.3000472.s027.png]
