# Supplementary material for: Building de novo cryo-electron microscopy structures collaboratively with citizen scientists
Source: PLoS Biol. 2019 Nov 12;17(11):e3000472. doi: 10.1371/journal.pbio.3000472 (PMC6850521; doi:10.1371/journal.pbio.3000472)
Supplement: S1 Text — (DOCX) [file pbio.3000472.s028.docx]

**S1 Text: Supplemental Results**

Between the four computational modeling approaches, Rosetta *denovo_density* had the greatest trouble, apparently due to Rosetta’s reliance on secondary structure predictions for backbone conformational sampling. Between the hand-built models, the backbone fitting was overall similar (S1 Table), although the Foldit structures consistently had better Ramachandran space geometry (Table 1). The differences in fitting are most apparent by comparing side chain fit, as the density was ambiguous for many side chains (Fig 1, S1-S6 Fig). Due to the maps’ resolution, the models from each method can vary slightly and still similarly fit the density, as the map versus model Fourier Shell Correlation (FSC) plots are very similar in both cases (Fig 1G, S8 Fig).

Multiple Foldit puzzles were given for these four cryo-EM targets. This first puzzle was posted during the CASP13 experiment (<http://predictioncenter.org/casp13/>), for the 149 residue-long CASP13 target T1021s1, where Foldit players were given five starting models predicted by 5 different CASP13 server predictions (MULTICOM-CONSTRUCT_TS1, QUARK_TS1, YASARA_TS1, Zhang-CEthreader_TS1, and Zhang-Server_TS1). Without any experimental data, Foldit players were unable to get close to the native state (S13A Fig). This was not surprising, as this protein has no obvious homologs, and none of the server predictions were close to the native fold.

The CASP13 organizers generously put the Foldit developers in contact with the experimentalists for this protein once the CASP13 experiment was over, and the above puzzle was reposted, this time with cryo-EM data. By providing the Foldit players with an electron potential map that is part of the score function—where they receive a score bonus for matching the density—players were easily able to match the native state (S13B Fig). As with all these Foldit puzzles, the native structure was unpublished and not available to the players.

This same process was repeated with CASP13 T1022s1, where the Foldit players were again initially given the same five server predictions for this different CASP target (MULTICOM-CONSTRUCT_TS1, QUARK_TS1, YASARA_TS1, Zhang-CEthreader_TS1, and Zhang-Server_TS1). Without any experimental data, the Foldit players did poorly. This protein was longer than the first case: 221 amino acids (S14A Fig). Despite this length, Foldit players reached the native state once they were given the cryo-EM density map (S14B Fig).

We then provided the players with two additional cryo-EM cases that were 149 residues and 114 residues long, respectively. These subunits were not used as targets for the CASP13 experiment, so we provided the Foldit players with no starting topology, just an extended polypeptide chain and the cryo-EM density map (S15 Fig).

Despite having to start with an extended chain, the players were able to quickly converge to the correct fold using the 3.2 Å cryo-EM map. S16 Fig shows the progression of the first 24 hours of folding (for the players who chose to play that day), followed by the first two days, in which some players had already reached the native state. By comparison, the microscopist spent a similar amount of time on each of these four targets: a first day to manually place all residues into the map, then the next day to loop over automated refinements and manual adjustments.

Note: The CASP assessors have been using the Global Distance Test (GDT_TS) score since 1998’s 3^rd^ CASP experiment^[[1]](#endnote-1)^.

1. Zemla A. LGA: A method for finding 3D similarities in protein structures. Nucleic Acids Res. 2003; 31(13):3370-4. DOI: 10.1093/nar/gkg571; PMID: 12824330; PMCID: PMC168977 [↑](#endnote-ref-1)
