# Supplementary material for: Building de novo cryo-electron microscopy structures collaboratively with citizen scientists
Source: PLoS Biol. 2019 Nov 12;17(11):e3000472. doi: 10.1371/journal.pbio.3000472 (PMC6850521; doi:10.1371/journal.pbio.3000472)
Supplement: S2 Text — (DOCX) [file pbio.3000472.s029.docx]

**Foldit Player Testimonials**

|  | **Top scoring solution** | **Top scoring Group share** | **Foldit Team** |
| --- | --- | --- | --- |
| **Puzzle 1572 (Afp1):** | matosfran | matosfran | Beta Folders |
| **Puzzle 1598 (Afp5):** | Galaxie | grogar7 | Anthropic  Dreams |
| **Puzzle 1588 (Afp7):** | isaksson | Bruno Kestemont | Go Science |
| **Puzzle 1606 (Afp9):** | Galaxie | Susume | Anthropic  Dreams |

**Puzzle 1572 (Afp1):** [**https://fold.it/portal/node/2005691**](https://fold.it/portal/node/2005691)

***matosfran*:** [**https://fold.it/portal/user/290222**](https://fold.it/portal/user/290222)

My overall strategy in design puzzles is usually to start by choosing the secondary structures according to the past most successful results and available protein length (along with other constraints). In the case of prediction puzzles such as 1572, I usually just rotate between the starting points and visually identify which one better matches the density cloud.

Following the rough identification of the secondary structure, and hand-placement both by dragging into a desirable place and using bands^[[1]](#endnote-1)^, I try to optimize the worst scoring backbones by either placing them in more desirable locations or fixing the structures by using the Remix tool^[[2]](#endnote-2)^, the Rama map^[[3]](#endnote-3)^ or by brute forcing the atoms into place with bands with help of the stick view.

Next, to avoid too much fine-tuning and try avoiding local minima (and to allow me to spend my time on something else), I switch over to recipes, generally GAB-based ones^[[4]](#endnote-4)^, which allow to explore a wide variety of solutions close to the one it started in whilst still providing some variety to allow the score to climb.

After allowing the recipe to do its magic for a while, I either try to manually improve the scoring (better placement of amino acids, changing the structure of some part, fixing something that became worse, etc.), or switch to other recipes, like DRW-based ones^[[5]](#endnote-5)^ which work more on a local basis. This routine is performed for a while with some Blue Fuse^[[6]](#endnote-6)^ sometimes spiced in (which I generally do manually to 10% clashing importance to watch if some parts improve or not, by comparing with a previously saved solution loaded back and coloring relative to that).

In the late-game, I leave it mainly to recipes, with some favorites being Acid Tweeker^[[7]](#endnote-7)^, Cut and Wiggle family^[[8]](#endnote-8)^, MicroIdealize^[[9]](#endnote-9)^ and walkers^[[10]](#endnote-10)^.

To sum-up, initially most of the time is dedicated to hand-folding (in Low Wiggle Power^[[11]](#endnote-11)^) whilst the recipes become progressively more prominent later in the folding process (together with increasing Wiggle Power to Medium). No resources and such are used outside of Foldit.

**Puzzle 1588 (Afp7):** [**https://fold.it/portal/node/2006093**](https://fold.it/portal/node/2006093)

***Bruno Kestemont*:** [**https://fold.it/portal/user/447652**](https://fold.it/portal/user/447652)

This puzzle started on 10/16/18 for 2 full weeks, which was very needed because ED puzzles need regular hand-folding sessions and are slow on computers.

From my files (local saves, S18A Fig), I can count that I intervened in this puzzle about 200 times. Interventions are 15-60 minute hand-folding (and/or 8-12h recipe) sessions, after which I save the result locally with a comment on what I did. As time passed, a synthetic “language” emerged within the group for these comments (S18A Fig). In addition, many recipes automatically add some information to the notes for segments, starting from the first available segment (S18B Fig).

Recipes are usually running in parallel on 3-5 parallel tracks, after which I share the best results (1 best score and 0-2 alternative best patterns). Additionally, I usually evolve in parallel the group’s best shared result. The advantage of working in parallel on different tracks is not only to be able to take the best solution from different tries, other tracks (including those from other players) inform us about successful recipe and subunit patterns.

We were allowed to load our results from previous puzzle 1579 (which had 5 server predictions and no density cloud). I saw that neither my best result from the previous round nor the proposed server models had any chance to fit into the density map. Team members shared the same impression on the group chat. This was confirmed by the researcher’s blog (S19 Fig).

I started a hard hand-fold from scratch. The key for starting these is to identify some aromatics in the cloud matching the ones in the protein. Fortunately, I could recognize some patterns on one end of the protein and progressively include them into the density cloud (S20 Fig).

After an hour of hand folding, I froze the rest of the protein and ran a rebuilding/idealizing recipe overnight (focusing on the already positioned segments). I shared this partly solved protein with my team in order to help them start their own solo runs.

Over the next days I used the same method, hand-folding a new piece when I found free time, fine tuning it with recipes 24/7 the rest of the time. For hand-folding, I progressively got inspiration from my group’s top scoring solution. The group’s top solution originated from an initial design by player *Batz* and was evolved by various players, including myself (S18 Fig). It took me 8 hand-folding sessions to finish fitting the protein into the cloud.

I started 5 other “hand fold” designs that had poor results, but the successful pieces I could fold from them were visually copied in the last score-gaining track. I also tried a 9^th^ fundamental hand fold, but at that time, recipes and small hand fold adjustments began to gain more on the 8^th^ version displayed in S21 Fig. The acronym of the mean recipes used from S21F Fig can be seen in the comments for “B2p8” in S18A Fig. From this share, the team also started to evolve the “B2p8” solution instead of *Batz*’s track.

The main “gaining” recipes used were:

-Tvdl enhanced DRW 2.8.1a^[[12]](#endnote-12)^ (edrwa), a group recipe for rebuilding/idealizing worst parts overnight

-Quickfix 3.5.5^[[13]](#endnote-13)^ a short-term recipe used during hand folding

-Jolter 2.0^[[14]](#endnote-14)^ (j) a structure rebuilding (loops, helices) recipe used during hand fold and overnight

-Quaking Remix 1.4.4^[[15]](#endnote-15)^ (qrmx) a remixing recipe used overnight

The other recipes mentioned in S17 Fig are Low Wiggle strategy recipes, for fine tuning during end games or fixing a desired design.

After the puzzle finished, some players continued evolving the group’s top solution. The latest “B2p8” solution (S22A Fig) shared by player *jeff101* scores 22,373 points four days after the expiration deadline (our final credited group score was by player *isaksson* with 22,359 points, my credited one scored only 22,343 points). One month after the deadline, *jeff101* shared the final gaining (but not credited nor used in this paper) solution, scoring 22,381 pts (S22B Fig).

***isaksson*:** [**https://fold.it/portal/user/719937**](https://fold.it/portal/user/719937)

My initial approach is to look at what the team have accomplished so far. I find it very difficult to find the initial fold. So, in the beginning I spend 90% of the time hand-folding, as no scripting will give you the expected result.

Eventually, when it is sort of more stable, there are many sections that needs to be changed. Here I use more common scripts, like "Constructor v1.05"^[[16]](#endnote-16)^ which I find very helpful. I also use other tools like "Fracture v2.1"^[[17]](#endnote-17)^, and "TvdL enhanced DRW 3.0.1"^[[18]](#endnote-18)^. Naturally, I used my own script "Mini Snake v1.0.9"^[[19]](#endnote-19)^.

What actually boosts my final score at the end of each puzzle, was to add some extra code/logic when running the above scripts concurrently. I had (my son has it now for gaming) a 6-core computer, and for that reason could run several scripts at the same time. After each round I would check the stored scores. If I find a higher score, then the current running script picks it up and uses that model instead as a base. I have about 3 different algorithms/scripts, times 2 as I also use different settings for the Wiggle power. So, now I have 6 different scripts competing in giving me the highest score. Each instance will compare its own score towards the highest stored one at the end of each round, and when each instance starts over for a new round it will always continue using the highest scoring instance instead, and so it continuous as long as I run the scripts.

I included some code on the next page, so you can get the idea of how it works. Below I have two functions "before" and "after". "Before" will check if there are higher scores to be picked up. "After" will check if it should store its result or not.

What I’d like to add is a tool to automatically remove stored instances. I currently have to remove them manually, before filling up the entire system, and that will normally be every 24h.

***isaksson’s* example code**

**function** **before_Solution**(rnd,str_name)

print("-Before-")

hi_score = **0**

hi_idx = **1**

sol = save.GetSolutions()

**if** (sol[**0**] == **nil**) **then**

print("0 solutions found")

--save.SaveSolution("auto_"..rnd)

**else**

--print((#sol+1) .. " solutions found")

**for** idx = **0**, #sol **do**

soli = sol[idx]

**if** (string.find([soli.name](http://soli.name/),str_name) ~= **nil**) **then**

--print([soli.name](http://soli.name/) .. " : " .. soli.score)

**if** (soli.score > hi_score) **then**

hi_score = soli.score

hi_idx = idx

**end**

**end**

**end**

--print("Load the solution ...")

**if**(current.GetEnergyScore()+save_resolution < hi_score) **then**

save.LoadSolution(sol[hi_idx])

soli = sol[hi_idx]

print("Load solution: "..[soli.name](http://soli.name/).." : "..rounding(soli.score,value_score_resolution))

**end**

**end**

**end**

**function** **after_Solution**(rnd,str_name)

print("-After-")

hi_score = **0**

hi_idx = **1**

sol = save.GetSolutions()

**if** (sol[**0**] == **nil**) **then**

print("0 solutions found")

str = "auto".."-"..rnd.."-"..rounding(current.GetEnergyScore(),value_score_resolution)

print("Save solution "..str)

save.SaveSolution(str)

**else**

--print((#sol+1) .. " solutions found")

**for** idx = **0**, #sol **do**

soli = sol[idx]

**if** (string.find([soli.name](http://soli.name/),str_name) ~= **nil**) **then**

--print([soli.name](http://soli.name/) .. " : " .. soli.score)

**if** (soli.score > hi_score) **then**

hi_score = soli.score

hi_idx = idx

**end**

**end**

**end**

--print("Save the solution ...")

band.DeleteAll()

**if**(current.GetEnergyScore()-save_resolution > hi_score) **then**

str = str_name.."-"..rnd.."-"..rounding(current.GetEnergyScore(),value_score_resolution)

print("Save solution: "..str)

save.SaveSolution(str)

**end**

**end**

**end**

**Puzzle 1598 (Afp5):** [**https://fold.it/portal/node/2006204**](https://fold.it/portal/node/2006204)

***grogar7*:** [**https://fold.it/portal/user/343874**](https://fold.it/portal/user/343874)

Puzzle 1598 was a Cryo-EM Electron Density puzzle. Players were presented with an extended chain of amino acids with no secondary structure indicated. In addition, we were given a high-resolution electron density (ED) map of the protein from cryo-electron microscopy data.

When I see this type of puzzle, the first things I look for are tryptophan residues in the chain, because the paddle shape of that amino acid’s side chain stands out fairly clearly in the ED map. This protein contains four tryptophans, at positions 53, 90, 108 and 118.

Next, I examine the ED map. Learning how to adjust the threshold and alpha (brightness) of the ED map is critical here, so one can distinguish various features of the map. I was able to see two of the distinctive paddle shapes fairly quickly and marked them with purple ‘note’ dots^[[20]](#endnote-20)^ (S2 Fig & S5 Fig, green model).

I looked at each of the tryptophan residues in the protein and examined what amino acids flanked them on either side. #53 is flanked by two leucines. #90 has an alanine on one side and a glutamine on the other. #108 has a threonine on either side of it. #118 is flanked by a glutamine and an arginine. Looking back at the ED map, the tryptophan marked by a purple dot on the long loop has identical looking sidechains flanking it and pointing opposite the tryptophan and toward each other. This observation ruled out #90 and #118, and made it very likely that #53 belonged here.

I think I used Phyre2 (a protein modeling program available online^[[21]](#endnote-21)^) to confirm that #53 was indeed on the long loop. So, I used the ‘Move’ tool^[[22]](#endnote-22)^ to place #53 in that position in the ED map oriented as it looked like it should be. I also noted carefully the placing of the protein backbone. I had a 50% chance of having it oriented in the right direction.

From this point, the process of placing the rest of the protein in the map was labor-intensive, painstaking hand-folding. I used the Rama Map tool^3^ to carefully manipulate each amino acid residue into its place in the ED map. Within a few amino acid placements on each side of the tryptophan, I was able to determine that the direction of the backbone was correct. The sidechains either maneuver into place or they don’t! Once I was certain I had the direction right, I continued maneuvering residues into place, one at a time, until the entire protein was placed. During the process I used the Wiggle^[[23]](#endnote-23)^ and Shake^[[24]](#endnote-24)^ tools fairly frequently to ‘settle’ sections of the protein into the map. I did this with Clashing Importance^[[25]](#endnote-25)^ (CI) set to 0.1 or 0.2 making the protein more flexible and Wiggle Power^11^ set to Low to increase speed. The entire process took 2 ½ days (12-16 hours of screen time). It was not always straightforward. Sometimes I made mistakes and had to rework several amino acid placements. Finding one end of the protein felt like a major accomplishment!

After the initial placement I used scripts to refine the protein. *Timo Vanderlaan*’s Tvdl enhanced DRW 3.0.2^[[26]](#endnote-26)^ is chief among these. This script performs many rebuilds on sections of the protein in an attempt to improve score. I used it at CI 0.2 and length 9-8 for around 24 hours. This script does a great job of fixing major problem areas of the protein and boosting the score. It can also introduce problems which require fixing by hand. When DRW stopped producing points, I reexamined the protein, fixed problem areas, and increased the CI to 0.4. I used Quaking Rebuild V2 1.0^[[27]](#endnote-27)^ for a couple of passes through the entire protein, then increased the CI to 1.0 and used B5 Compressor Gentle NC 2/30^[[28]](#endnote-28)^ to further refine the protein and increase score. Keeping the Wiggle Power at Low and CI at 1.0, I used Tvdl enhanced DRW 3.0.2 again, but with shorter lengths (5-2) for more refinements. I also used another banding script, Rav3n_pl GAB v3.4.10 filter Cuts^[[29]](#endnote-29)^ by *LociOiling* before increasing the Wiggle Power to Medium (which was the highest available on this puzzle). Finally, I used Walkin’ Rebuild v4.0.4 nc 25^[[30]](#endnote-30)^ and Banded Worm Pairs Inf Filt 1.4.3^[[31]](#endnote-31)^ until the puzzle expired.

I am frequently in the top 15 players on a puzzle, using the method described, but have only rarely won top place. Of course, finishing first is always exhilarating!! I have been playing Foldit since October of 2011 and have not tired of the challenge yet!

**Testimonial from top-scoring player on both Puzzle 1598 (Afp5) and Puzzle 1606 (Afp9)**

***galaxie*:** [**https://fold.it/portal/user/352715**](https://fold.it/portal/user/352715)

One of the advantages of being on a team in the Foldit community is the ability to share and improve protein structures as a group project. Our team has several players who excel at setting up a chain of amino acids in an electron density (ED), defining secondary structure (sheets and helices) in the process. These players (*soloists*) share their solutions with the rest of the team, usually somewhere between mid and end game. Teammates who continue to refine and develop these shares are called *evolvers*.

As an *evolver* I look at the description of the work done by the teammate posting a share. Usually with ED puzzles the backbone and most of the sidechains are well placed when the protein structure is shared. Small segment rebuilders (such as Tvdl enhanced DRW^26^) and banders (banded worm pairs^31^) are used to settle the protein in the density. Sometimes using a band to manually pull sidechains can help fit these sidechains into their respective ED "pockets".

ED puzzles can be very challenging and extremely rewarding. Working on this type of puzzle gives players a better appreciation for structures that are well placed in the density.

**Puzzle 1606 (Afp9):** [**https://fold.it/portal/node/2006275**](https://fold.it/portal/node/2006275)

***Susume*:** [**https://fold.it/portal/user/341210**](https://fold.it/portal/user/341210)

I started by getting the sequence of amino acids (using the AA print recipe^[[32]](#endnote-32)^) and putting them in a text document. I marked all the aromatics (the ring sidechains) in the sequence, and noticed which ones had potentially recognizable neighbors. For example, residues 11-13 are WAF - two aromatics with a small alanine between them. 53 is a Y followed by 3 glycines (which have no sidechain) - that might be recognizable in the cloud. 58-62 are HDFIF - three aromatics close together, with an I in between them - because I's sidechain is asymmetrical it can sometimes be recognized in the cloud.

I also got the secondary structure prediction (using the SS print recipe^[[33]](#endnote-33)^) and printed that under the sequence in the text document. I noted that 40F and 84H were both aromatics that were predicted to be at or near the end of a helix.

Then I placed the protein (still a straight line) in the cloud and started moving it around and zooming in on different spots to look for any recognizable details. It's just not possible to see things in the cloud unless you have some protein in there for perspective, even if it is an unrelated section of the protein. You just have to get something in there to use as a visual reference.

As I move around looking for details in the cloud (moving the straight protein around as I go to bring different parts of the cloud into focus), I mark the ends of helices (red dots) and sheets (blue dots) if I can see any. I also mark aromatic sidechains (purple dots). When I find and mark an aromatic, I look at the sidechain pockets on either side along the backbone to see if I can identify anything about them - small? large? missing? another aromatic? Then I compare that little sequence of details in the cloud to the aromatics I marked in the text document.

In this protein the first toehold I got was seeing three aromatic sidechain pockets close together on the outside of the cloud. I placed 58-62 HDFIF (which I had noticed earlier in the sequence) in that spot in the cloud, using the I at 65 to decide which direction to turn them.

From there I worked toward the higher numbered residues, because there is a long helix just after those aromatics that was pretty easy to place. I worked slowly along the backbone, using a pin to hold my finished work in place as I went. I temporarily changed the SS on upcoming residues to sheet or helix so I could use "idealize SS"^[[34]](#endnote-34)^ to give them a starting shape that seemed similar to the cloud. Then I used the Rama Map^3^ to adjust the sheets or helices and to bend the loops into the shape dictated by the cloud. On some proteins I have to insert a lot of cut points^[[35]](#endnote-35)^ as I go along because the Rama Map adjustments get further and further away from the cloud, but this protein required very few cut points (just eight). Once I got to the end, I went back to 58-62 and worked from there to the beginning.

As I go, I rubber band^1^ many of the beta carbons (the first joint in the sidechain) to the place I think they will go in the cloud. It's easier to see where that first sidechain joint goes than to see where the backbone carbons will go, and I feel like beta carbon bands constrain how the backbone twists more than backbone bands do (so they force the backbone more specifically to the shape I intend).

On some proteins I will freeze the part I have not yet placed and shake/wiggle what I have placed to settle it a bit before continuing. On others I just place it all as best I can before ever doing a wiggle. I don't like to let wiggle move the backbone at all on sections I have not yet matched to the cloud - I would much rather work from the extended chain than from some random shape that wiggle got by pulling the unmatched parts into nearby unrelated parts of the cloud.

Once I had the whole protein placed, I shook and wiggled for the first time at low CI, then closed all the cut points. After another wiggle I used a Cut & Wiggle^8^ script for one iteration to fix ideality where the backbone was stretched or compressed from my earlier cut points. Then I ran my usual sequence of scripts. On some ED puzzles I will stop after one or two scripts and use hand work to try to better match the cloud where the match is poor, but on this one the scripts seemed to be pulling things into good alignment so I omitted more hand work. When it looked like I might have a high-ranking solo, I shared it with my team. From there *Galaxie* was able to improve it even more; see previous section: *Testimonial from top-scoring player on both Puzzle 1598 (Afp5) and Puzzle 1606 (Afp9)*.

1. <https://foldit.fandom.com/wiki/Band> [↑](#endnote-ref-1)
2. <https://foldit.fandom.com/wiki/Remix> [↑](#endnote-ref-2)
3. <https://foldit.fandom.com/wiki/Rama_Map> [↑](#endnote-ref-3)
4. <https://foldit.fandom.com/wiki/GAB> [↑](#endnote-ref-4)
5. <https://foldit.fandom.com/wiki/DRW> [↑](#endnote-ref-5)
6. <https://foldit.fandom.com/wiki/Fuse> [↑](#endnote-ref-6)
7. <https://doi.org/10.1073/pnas.1115898108> [↑](#endnote-ref-7)
8. <https://fold.it/portal/recipe/48874> [↑](#endnote-ref-8)
9. <https://fold.it/portal/recipe/48411> [↑](#endnote-ref-9)
10. <https://foldit.fandom.com/wiki/Local_Wiggle_Strategy> [↑](#endnote-ref-10)
11. <https://foldit.fandom.com/wiki/Wiggle_Power> [↑](#endnote-ref-11)
12. <https://fold.it/portal/recipe/101258> [↑](#endnote-ref-12)
13. <https://fold.it/portal/recipe/100698> [↑](#endnote-ref-13)
14. <https://fold.it/portal/recipe/101947> [↑](#endnote-ref-14)
15. <https://fold.it/portal/recipe/102099> [↑](#endnote-ref-15)
16. <https://fold.it/portal/recipe/46192> [↑](#endnote-ref-16)
17. <https://fold.it/portal/recipe/101250> [↑](#endnote-ref-17)
18. <https://fold.it/portal/recipe/49233> [↑](#endnote-ref-18)
19. <https://fold.it/portal/recipe/102841> [↑](#endnote-ref-19)
20. <https://foldit.fandom.com/wiki/Electron_Density_Puzzles> [↑](#endnote-ref-20)
21. <http://www.sbg.bio.ic.ac.uk/phyre2/html/page.cgi?id=index> [↑](#endnote-ref-21)
22. <https://foldit.fandom.com/wiki/Move> [↑](#endnote-ref-22)
23. <https://foldit.fandom.com/wiki/Wiggle> [↑](#endnote-ref-23)
24. <https://foldit.fandom.com/wiki/Shake> [↑](#endnote-ref-24)
25. <https://foldit.fandom.com/wiki/Clashing_Importance> [↑](#endnote-ref-25)
26. <https://foldit.fandom.com/wiki/EDRW> [↑](#endnote-ref-26)
27. <https://fold.it/portal/recipe/35801> [↑](#endnote-ref-27)
28. <https://fold.it/portal/recipe/48095> [↑](#endnote-ref-28)
29. <https://foldit.fandom.com/wiki/GAB> [↑](#endnote-ref-29)
30. <https://fold.it/portal/recipe/48198> [↑](#endnote-ref-30)
31. <https://fold.it/portal/recipe/100682> [↑](#endnote-ref-31)
32. <https://fold.it/portal/recipe/45037> [↑](#endnote-ref-32)
33. <https://fold.it/portal/recipe/102495> [↑](#endnote-ref-33)
34. <https://foldit.fandom.com/wiki/Idealize_SS> [↑](#endnote-ref-34)
35. <https://foldit.fandom.com/wiki/Cutpoints> [↑](#endnote-ref-35)
