# Supplementary material for: Building de novo cryo-electron microscopy structures collaboratively with citizen scientists
Source: PLoS Biol. 2019 Nov 12;17(11):e3000472. doi: 10.1371/journal.pbio.3000472 (PMC6850521; doi:10.1371/journal.pbio.3000472)
Supplement: S1 Authors — (DOCX) [file pbio.3000472.s030.docx]

**The following players have declared no competing interests:**

Alan Coral

Alexander Boykov

Alexander Paul Brough

Allen Richard Lubow

Alyssa Joy Higgins

Дмитрий Сергеевич Голощапов

Bartosz Kaszubowski

Binyou Wang

Brett A. Burkholder

Brian M. Lee

Bruno Kestemont

Catherine R Landers

Charles Coqueret

Charles Cusack

Charles David Coleman

Cheryl Greenwell

Christiaan Narinx

Christopher D. Eastlake

Coby Walker

Dahlia Dry

Dave Trownsell

David Murray

Douglas Craig Wheeler

Ernest Webb

Gary O. Gross

George Victor McIlvaine

Gregory T. Hansen

Harald Feldmann

Heidemarie Karin Fuentes

Istvan Kovanecz

Jake Fisher

Jami Lynne Borman

Jasper A. Diderich

Jeffrey M Canfield

Joanne Mitchell

John Michael Joseph McMahon

John Mitch

Jonas Schinkler

June M Brownlee

Justin Wangying Lam

Keith T. Clayton

Kenneth E. DeFord

Kevin Dale Wells

Kirk Joseph Abbey

Larry C. Withers

LaVerne Poussaint

Leanne Riki Cheever

Lennart E. Isaksson

Linda Wei

Luis Alonzo Villagomez

Lynn Carpenter

Manasa Sharma

Marco Post

Michael Krapukhin

Patrick Brady

Patrick John Rabaja Camarador

Pavel Varlashin

Pavlo Denys

Peter John Triggiani IV

Ricardo Oliveira da Silva

Robert Gamble

Robert Leduc

Roman Madala

Savas Pashalis

Scott Shnider

Sebastian Graf von Matuschka

Sergey Pinyaev

Stefan Kunert

Steven A. Schwegmann

Susan Curry Kleinfelter

Sven Michael Holst

Thomas Bausewein

Thomas J. George

Timotheus J.A. van der Laan

Todd Burckin

Ulas Yeginer

Usman Rehman

Vasiliy Sidoruk

Vedran Sabljak

Vera Simon

Walter Barmettler
